# Generation and Characterization of Germline-Specific Autophagy and Mitochondrial Reactive Oxygen Species Reporters in *Drosophila*

**DOI:** 10.3389/fcell.2019.00047

**Published:** 2019-04-03

**Authors:** Kiran Nilangekar, Nidhi Murmu, Govind Sahu, Bhupendra V. Shravage

**Affiliations:** ^1^Developmental Biology Group, Agharkar Research Institute, Pune, India; ^2^Department of Biotechnology, Savitribai Phule Pune University (SPPU), Pune, India

**Keywords:** GFP-Ref(2)P, mCherry-Atg8a, mito-roGFP2-Orp1, autophagic flux, redox, germline stem cell, autophagy, Atg8a

## Abstract

Oogenesis is a fundamental process that forms the egg and, is crucial for the transmission of genetic information to the next generation. *Drosophila* oogenesis has been used extensively as a genetically tractable model to study organogenesis, niche-germline stem cell communication, and more recently reproductive aging including germline stem cell (GSC) aging. Autophagy, a lysosome-mediated degradation process, is implicated in gametogenesis and aging. However, there is a lack of genetic tools to study autophagy in the context of gametogenesis and GSC aging. Here we describe the generation of three transgenic lines mcherry-Atg8a, GFP-Ref(2)P and mito-roGFP2-Orp1 that are specifically expressed in the germline compartment including GSCs during *Drosophila* oogenesis. These transgenes are expressed from the *nanos* promoter and present a better alternative to UASp mediated overexpression of transgenes. These fluorescent reporters can be used to monitor and quantify autophagy, and the production of reactive oxygen species during oogenesis. These reporters provide a valuable tool that can be utilized in designing genetic screens to identify novel regulators of autophagy and redox homeostasis during oogenesis.

## Introduction

In multicellular organisms, the production of functional gametes depends on the activity of specialized stem cells called “germline stem cells” (GSCs) located in the gonads (reviewed in Fuller and Spradling, 2007; Dansereau and Lasko, 2008). Like most other types of stem cells, GSCs are also subject to cellular damage that causes premature aging and render them inactive leading to their depletion within the gonads (López-Otín et al., 2013; Signer and Morrison, 2013; Oh et al., 2014). Cellular damage caused by genotoxic agents and reactive oxygen species can damage organelles, proteins, and DNA within the stem cells. Thus, it is crucial to maintaining the integrity and quality of GSCs, as cellular and genetic defects may be passed onto the next generation which can be detrimental to the survival of the species. The prolonged sustenance of GSCs, therefore, require efficient homeostasis mechanisms to be operational constitutively within these cells. Such homeostasis mechanism(s) must function to significantly reduce cellular damage. Macro-autophagy (autophagy) is the cellular mechanism that is involved in removing toxic protein aggregates and damaged organelles such as mitochondria from within the cytoplasm (Takeshige et al., 1992; Mizushima, 2007; Yu et al., 2018). Autophagy is necessary during animal development and impaired autophagy has been implicated in several diseases including cancer, neurodegenerative diseases, infectious diseases, cardiopathy and autoimmunity (Jiang and Mizushima, 2014; Schneider and Cuervo, 2014). However, the involvement of autophagy during gametogenesis, reproduction and germline aging is not extensively studied.

Autophagy proceeds with the sequestration of cytoplasmic contents within a double-membraned vesicle termed as autophagosome which fuses with the lysosome to form an autolysosome. The cargo within the autolysosome is degraded by the lysosomal enzymes. The degradation products are exported from the autolysosome to the cytoplasm where they can be reused in several metabolic processes (He and Klionsky, 2009; Boya et al., 2013; Bento et al., 2016). The complex process of autophagosome formation and its subsequent fusion to the lysosome is highly regulated by Autophagy-related (Atg) proteins (Mizushima et al., 1998, 2011; Klionsky, 2012; Mulakkal et al., 2014).

In metazoans, 16 Atg proteins have been shown to be essential for the autophagy. The process is initiated at the pre-autophagosome structure/phagophore assembly site (PAS) where Atg1 kinase complex consisting of Atg1, FIP200, Atg13, and Atg101 assembles (reviewed in Scott et al., 2004; Nagy et al., 2014b; Hurley and Young, 2017). PAS formation can occur at the ER and is termed as omegasome in mammals (Axe et al., 2008). The activity of Atg1 kinase is required for recruitment of Vps34 complex comprising of Beclin-1 (Atg6 in *Drosophila*), Vps34, Vps15 and Atg14 to the phagophore where it catalyzes the formation of PI3P which is essential for vesicle nucleation (Kihara et al., 2001; Itakura et al., 2008; Juhász et al., 2008). Several membrane sources can contribute to the formation of the phagophore/isolation membrane (Chan and Tang, 2013). The formation of Phosphatidylinositol 3-phosphate (PI3P) at the phagophore site is crucial for the recruitment of WIPI family proteins (Burman and Ktistakis, 2010). WIPI2 (Atg18 in *Drosophila*) family proteins interact with Atg16L1 (Atg16 in *Drosophila*) that subsequently allow for recruitment of Atg12-Atg5-Atg6L1 complex at the PAS (Romanov et al., 2012; Walczak and Martens, 2013). The formation of the Atg12-Atg5-Atg16 complex is catalyzed by several enzymes. Atg12 is a ubiquitin-like protein that is activated by Atg7 E1-like enzyme and subsequently transferred to E2 like enzyme Atg10 allowing for the formation of Atg12-Atg5 complex. The Atg12-Atg5 complex can form a ternary complex with Atg16L1 (Atg16 in *Drosophila*), finally catalyzing lipidation of Atg8 on the autophagosome membrane (Mizushima et al., 1998; Kim et al., 2016; Nagy et al., 2017). Atg8/MAP1LC3, a ubiquitin-like molecule (microtubule associated protein 1 light-chain 3, LC3, in mammals) is a component of conjugation system that is also essential for elongation and completion of the autophagosome (Ichimura et al., 2000; Kabeya et al., 2000). C-terminal amino acid residues of Atg8 are removed by ATG4 protease to expose a C-terminal glycine that is covalently bound to phosphatidylethanolamine (PE) by series of enzymes Atg7 (E1-like enzyme), Atg3 (E2-like enzyme), and the Atg12 complex (consisting of Atg12-Atg5-Atg16). The Atg8-PE (Atg8-II) is localized to the autophagosome membrane and this lipidation event has been exploited to study autophagy. Typically, Atg8 is fused with fluorescent proteins such as GFP or RFP/mCherry at its amino terminus and are driven by tissue-specific promoters, endogenous Atg8a promoters, by UAS and UASp sequences (Scott et al., 2004; Denton et al., 2009, 2012; Nelson et al., 2014; Hegedus et al., 2016; Jacomin and Nezis, 2016; Bali and Shravage, 2017). However, a few of these transgenes do not express in the germline or are expressed at very high levels in the germline complicating autophagy assays. An alternative to monitoring autophagy is to assay for lipid conjugation of Atg8 proteins by immunoblotting (Barth et al., 2011; Mauvezin et al., 2014; Nagy et al., 2015; Lörincz et al., 2017). The lipidated protein Atg8-II migrates at a faster rate on polyacrylamide gels than its unprocessed form, Atg8-I. However, immunoblotting techniques typically use protein extracts prepared from the entire tissue or group of cells which eliminate individual differences in autophagy status in individual cell types within the tissue (Klionsky et al., 2016).

p62/SQSTM1 is an autophagy adaptor first described in mammals and subsequently shown to function during autophagy in *Drosophila* (Ref(2)P in *Drosophila*) (Bartlett et al., 2011). The ability of p62/Ref(2)P to interact with ubiquitin or polyubiquitin chains in different proteins via the UBA domain enables it to deliver cargo to autophagosome via the LIR domain (which is also known as the Atg8 interacting domain in *Drosophila*). Interestingly, p62 is capable of both homo-oligomerization and hetero-oligomerization via PB1 domains thus allowing the formation of complex protein aggregates that can be recognized by the autophagy machinery and targeted for degradation (Mulakkal et al., 2014; Nagy et al., 2015; Lörincz et al., 2017). Ref(2)P has been used to measure autophagic activity in *Drosophila* fat body cells where an increase in the size of Ref(2)P punctae under stress conditions was observed followed by a reduction in number due to the clearance of protein aggregates by autophagy (Pircs et al., 2012). The increased levels of Ref(2)P which marks the protein aggregates during the inhibition of autophagy serves as an *in vivo* measure of long term autophagic activity. Thus, it can be used as a tool to detect the protein inclusion formation and changes in autophagic activity under various physiological conditions (Bartlett et al., 2011; Pircs et al., 2012; Devorkin and Gorski, 2014; Mauvezin et al., 2014). In *Drosophila*, there are no transgenic lines that can detect Ref(2)P in GSCs and any assays performed in the germline are based on the use of anti-Ref(2)P antibody which becomes a limiting factor for designing forward genetic screens.

The elongation of phagophore is catalyzed by Atg9/Atg9L1 complex in addition to the Atg1 and Vps34 complexes, and Atg12 and Atg8 conjugation systems. The Atg9 complex consists of Atg9, a transmembrane protein, Atg18, and Atg2 (Nagy et al., 2014a). Atg9 containing vesicles shuttle between the PAS/phagophore and membrane source (Golgi/endosomes). Membrane scission occurs at the end of the elongation process resulting in the generation of a closed autophagosome laden with cargo. Following this event, most Atg proteins are removed or disassembled from the autophagosome membrane catalyzed by PI3-P phosphatases which aid in the elimination of PI3-P from the membrane. In most metazoans, autophagosomes fuse with endosomes to form amphisomes before being fused to lysosomes by kiss-and-run like process. Several proteins which are involved in vesicle fusion including SNAP29, Syntaxin17, Rab7, Atg14, HOPS complex and PLEKHM1 are shown to be required for this process (Takáts et al., 2013; Guo et al., 2014; Takats et al., 2014; Liu et al., 2015; McEwan et al., 2015; Hegedus et al., 2016). The outer membrane of the autophagosome fuses to the lysosome to form the autolysosome. The cargo along with the inner membrane of the autophagosome is degraded in the autolysosome by the lysosomal hydrolases. The degradation products are exported from the autolysosome back into the cytoplasm where they are used for anabolic processes (Mizushima, 2007; Bento et al., 2016; Nakamura and Yoshimori, 2017; Yu et al., 2018).

Autophagy is also triggered in response to reactive oxygen species (Scherz-Shouval and Elazar, 2011; Filomeni et al., 2015). Hydrogen peroxide (H_2_O_2_), one of the metabolites of the intracellular redox reaction, is an important signaling molecule, is required for peroxisomal catabolism and used by cells in a controlled manner to oxidize substrates. However, it is also the main source of peroxide ions which oxidize and damage proteins, lipids, and DNA (Sies, 2017). One of the main sources of H_2_O_2_ is the mitochondrial electron transport chain (Dröge, 2002; Sena and Chandel, 2012; Shadel and Horvath, 2015). Superoxide ions O_2_^-^ are produced by electron transport chain complex I and III present in different compartments of the mitochondria. O_2_^-^ ions are reduced by superoxide dismutase (SOD) enzymes. Mn-dependent SOD2 in the mitochondrial matrix is responsible for the conversion of O_2_^-^ to H_2_O_2_. H_2_O_2_ is detoxified to water by catalase and glutathione peroxidase (Dröge, 2002). However, H_2_O_2_ can be damaging when it reacts with the thiol group (-SH) within the proteins to form sulphenic acid (-SOH) altering their activity or rendering them inactive (Finkel, 2012). Such cellular damage within mitochondria leads to depolarization of mitochondria which results in a decrease of ATP synthesis and metabolic activities. Multiple proteins within the cells have been demonstrated to sense production of H_2_O_2_ and react with it (Ma et al., 2007; Gutscher et al., 2008).

Oxidant receptor peroxidase 1 (Orp1), a component of redox relay in yeast *Saccharomyces cerevisiae*, is homologous to glutathione peroxidase and senses H_2_O_2_ (Ma et al., 2007). Orp1 is shown to be able to successfully oxidize roGFP2 based on H_2_O_2_ sensing *in vitro*. This was first demonstrated by Gutscher et al. (2008), by making an Orp1-roGFP2 fusion protein. This construct was then modified by adding a mitochondrial localization signal (mito-roGFP2-Orp1) by Albrecht and colleagues enabling measurement of H_2_O_2_ production within the mitochondria (Albrecht et al., 2011, 2014). The probe senses the redox state of Orp1 through roGFP2. Thiol groups in Orp1 are oxidized by H_2_O_2_. This alters the redox state of Orp1 that causes reduction or oxidation of the roGFP2 which results in the switch in excitation of fluorescence from 488 to 405 nm. The shift in excitation of roGFP2 is a reliable measure of the H_2_O_2_ production.

Here we describe generation and characterization of transgenic lines that express mCherry-Atg8a, GFP-Ref(2)P and mito-ro-GFP2-Orp1 under the *nanos* gene promoter. Our data show that these transgenes could be used reliably to monitor autophagy and H_2_O_2_ production within the germ cells in *Drosophila* during gametogenesis. These transgenes lack the problems associated with UASp driven transgenes which can overexpress the Atg proteins complicating and affecting the interpretation of autophagy. We believe that these transgenes will aid in conducting screens designed to identify genes affecting autophagy and redox status within the germ cells in *Drosophila*.

## Materials and Methods

### Fly Maintenance

All the transgenic fly stocks were maintained at 25°C on standard cornmeal sucrose malt agar. The stocks were maintained homozygous for all transgenes. All insertions were tested for expression of the transgene using confocal microscopy. Insertion with the highest expression (strong fluorescence) was chosen for characterization. mCherry-Atg8a and GFP-Ref(2)P were mapped on to the chromosome, balanced and crossed to get the following combination; ywhsFLP1; mCherry-Atg8a/CyO; GFP-Ref(2)P/TM6b. The following flies were used; yw; UASp.mCherry.Atg8a; Dr/ TM3, Ser (BL37750, RRID:BDSC_37750), w; +; nosGal4VP16 (BL4937, RRID:BDSC_4937), y sc v; +; Atg8a-RNAi (BL34340, RRID:BDSC_34340), y sc v; +; Atg5-RNAi (BL 34899, RRID:BDSC_34899), y Atg8a^KG07569^/FM7c; +; + (BL14639, RRID:BDSC_14639).

### Feeding, Starvation and Pharmacological Treatments

For each treatment, 24–96 h old flies in a group of 15 females and 10 males were housed in a vial.

#### Feeding

Flies were transferred every day into a fresh vial containing cornmeal sucrose malt agar supplemented with yeast paste/pellets.

#### Starvation

Flies were starved on vials containing 20% sucrose in 2.5% agar.

#### Chloroquine Treatment

Flies were transferred every day into a vial containing sucrose agar with chloroquine to a final concentration of 3 mg/ml.

#### Rapamycin Treatment

Rapamycin was added into food just before pouring food (60°C) to a final concentration of 200 μM.

To determine the optimal time of starvation where most number of mCherry-Atg8a punctae would occur (Supplementary Figure S3D), nosP-mCherry-Atg8a line was subjected to starvation for 1, 2, 3, and 4 days by transferring them daily into a vials containing 20% sucrose in 2.5% agar. nosP-GFP-Ref(2)P flies after being exposed to pharmacological treatments were dissected in Grace’s medium and imaged immediately on Nikon SMZ 1270 microscope fitted with Nikon DS-Fi2 camera. All images were acquired at the same magnification (Supplementary Figure S1C).

### Cloning of pC4 nosP-nos 3′UTR

nanos3′UTR was PCR amplified from CantonS genome using primers nos3′UTRF25′-tctagaagagggcgaatccagctctggagcaga and nos3′UTRR 5′-tctagaccattttgggagacgccttgaacctaagtg and digested with XbaI and PstI. The resulting fragment of 1236 bp was cloned in XbaI, PstI digested pCasper4 to obtain pC4-nos3′UTR. Nanos promoter was amplified using primers nosPF 5′-aagcttcgaccgttttaacctcgaaatatg and newnosPR 5′-tggcgaaaatccgggtcgaaagttacg to obtained 935 bp fragment. This fragment was cloned in pGEMt-Easy to obtain pGEMt-nosP. pGEMt-nosP was digested using EcoRI and the resulting 963bp fragment was cloned in EcoRI digested pC4-nos3′UTR to obtain pC4-nosP-nos3′UTR.

### nosP-GFP-Ref(2)P-nos3′UTR

2537 bp NotI-XbaI fragment from pUASt-GFP-Ref(2)P (Chang and Neufeld, 2009) was cloned in NotI-XbaI digested pC4-nosP-nos3’UTR to generate pC4-nosP-GFP-Ref(2)P-nos3’UTR.

Chang and Neufeld, 2009 have referred pUASt-GFP-Ref(2)P as GFP-Ref(2)P in their published article. However, GFP-Ref(2)P is generated using EGFP sequence from pEGFP (Clonetech, United States; Scott et al., 2004).

### nosP-mCherry-Atg8a-nos3′UTR

1162 bp XbaI fragment from pmCherry-Atga (Bali and Shravage, 2017) was cloned in XbaI digested pC4-nosP-nos3′UTR to generate pC4-nosP-mCherry-Atg8a-nos3′UTR.

### nosP-mito-roGFP2-Orp1-nos3′UTR

1241 bp NotI-XbaI fragment from pUASt-mito-roGFP2-Orp1 (Albrecht et al., 2011, 2014) was cloned in NotI-XbaI digested pC4-nosP-nos3′UTR to generate pC4-nosP-mito-roGFP2-Orp1-nos3′UTR.

All constructs were sequenced (1st Base, Malaysia), the sequences analyzed for accuracy and were verified to match the published sequence. Transgenic lines were generated at C-CAMP, Bangalore, India, using standard microinjection techniques.

### Fat Body Dissection

Third instar larvae were floated using 20% sucrose and transferred to a Petri dish. The larvae were dissected in 1xPBS, the cuticle was torn along the length of the larvae using forceps to expose all the internal organs including the fat body.

### Immunostaining

Third instar larvae were dissected in 1xPBS and fixed with 4% paraformaldehyde (PFA) overnight at 4°C. Fixed larval carcasses were washed with 0.1% PBTx (1xPBS + 0.1% Triton X-100) four times for 5 min each and blocked in PBTxGS (0.1% PBTx + 5% Normal Goat Serum) for 2–4 h at RT. After blocking, carcasses were incubated in primary antibody solution overnight at 4°C. The next day samples were washed with 0.1% PBTx four times for 20 min each and blocked in PBTxGS for 1–2 h at RT. Carcasses were incubated in secondary antibody solution for 2–3 h at RT (protected from light) followed by four washes with 0.1% PBTx for 20 min each. One μg/ml DAPI solution prepared in 0.1% PBTx was added to the sample and incubated for 10 min followed by washing with 0.1% PBTx three times for 10 min each. In the final step, the fat body along with the larval gonads was separated from the carcasses and mounted in Prolong Gold anti-fade reagent (Invitrogen Inc, United States).

Dissected adult ovaries were fixed in 4% PFA for 15 min, washed three times with 0.1% PBTx for 5 min each and blocked with 1% PBTx containing 0.5% BSA for 1 h at room temperature. The ovaries were incubated with primary antibody in 0.3% PBTx containing 0.5% BSA overnight at 4°C. The next day primary antibody was removed and the sample was washed with 0.1% PBTx for 15 min then blocked with 10% NGS in 0.1% PBTx for 2 h at room temperature. Ovaries were then stained with secondary antibody in 0.1% PBTx containing 10% NGS for 2 h at room temperature protected from light. Excess antibody was removed by three washes with 0.1%PBTX for 15 min each. One μg/ml DAPI solution in 0.1% PBTx was added to the samples and incubated at room temperature for 10 min followed by washing with 0.1% PBTx solution 3 times for 5 min each. Ovarioles were mounted in Prolong Gold.

The following antibodies and dilutions were used; anti-Cathepsin L (Abcam Cat# ab58991, RRID:AB_940826) 1:400 for larval tissues and 1:300 for adult ovaries; anti-ATP5α 1:100 (Thermo Fisher Scientific Cat# 43-9800, RRID:AB_2533548), anti-GABARAP 1:100 (Cell Signaling Technology Cat# 13733), anti-Ref(2)p 1:1000 (Abcam Cat# ab178440), anti-GFP 1:10 (DSHB Cat# DSHB-GFP-12A6, RRID:AB_2617417) anti-α spectrin 1:20 (DSHB Cat# 3A9 (323 or M10-2), RRID:AB_528473). Secondary antibodies used were Alexa fluor 555 goat anti-rabbit 1:250 (Molecular Probes Cat# A-21429, RRID:AB_141761) and Alexa fluor 488 goat anti-rabbit 1:250 (Molecular Probes Cat# A-11070, RRID:AB_142134).

### Redox Chemical Treatment

Ovaries of mito-roGFP2-Orp1 flies were dissected in Grace’s medium and washed in 1xPBS for 2 min. The ovaries were incubated in 4 mM diamide (Sigma Cat # D3648) in 1xPBS in order to oxidize or 40 mM DTT (SRL Cat# 17315) in 1xPBS in order to reduce for 10 min at room temperature with gentle shaking. They were washed once in 1xPBS for 2 min before being treated with the redox conservative reagent; 20 mM *N*-ethyl maleimide (Sigma Cat# E3876) in 1xPBS for 10 min at room temperature. The untreated set and pharmacologically treated samples were proceeded directly for redox conservation. They were washed once in 1xPBS before being fixed in 4% PFA for 15 min at room temperature. Following fixation, two washes of 1xPBS for 5 min each were given. The ovaries were mounted in 80% glycerol and imaged the same day. The redox treatment procedure is modified from Albrecht et al., 2011; (Nilangekar and Shravage, 2018).

### Imaging and Analysis

All imaging was performed on Leica SP8 Confocal microscope using 63x oil objective. Images acquired were 8 bit, 1024 × 1024 pixel resolution at 100 Hz scanning. Frame accumulation was performed with 6 frames for mCherry-Atg8a and GFP-Ref(2)P. Images were analyzed using ImageJ. For mCherry-Atg8a mean intensity measurements, an ROI was drawn around the GSCs, identified by the location and size of their nuclei. mCherry-Atg8a and GFP-Ref(2)P punctae were counted manually and the area of germarium were measured using ImageJ. For colocalization analysis, JACoP plugin was used (Bolte and Cordelières, 2006). Both the channels were thresholded in the plugin and Pearson’s coefficient were recorded. Microsoft Excel was used for statistical analysis. Student’s *T*-Test of two samples assuming unequal variance was performed for all comparisons. Graphs were plotted in GraphPad Prism 7.

### Imaging and Image Analysis of Mito-roGFP2-Orp1

roGFP2 was excited at 405 and 488 nm “line by line” and its emission from both of these excitations was collected between 500 to 530 nm. Frame accumulation was performed with 6 frames for both the channels.

The ratiometric analysis was performed using ImageJ by the following steps; the background was subtracted (roll ball = 50 pixels), images were converted to 32 bit, the 488 channel was thresholded and the background pixels were set as “NaN,” the intensity in the GSCs from the same region in both channels was measured. To generate the ratio image, “Ratio Plus” plugin was used and the resultant image was displayed in the “Fire” lookup table.

### Western Blotting

For lysate preparation, 25–30 flies were dissected in a buffer containing 150 mM NaCl, 25 mM Tris and protease inhibitors (Merck Cat # 11697498001). The ovaries were transferred to 1.5 ml tube and the buffer was replaced with RIPA buffer (150 mM NaCl, 25 mM Tris, 1% Nonidet-P 40, 0.5% Sodium deoxycholate, 0.1% SDS) containing protease inhibitors. The tissue was homogenized on ice using plastic pestles. The homogenate was centrifuged at 20,000 rcf for 3 min at 4°C, the supernatant was transferred to a fresh tube and this step was repeated two more times. This lysate was quantified using BCA (Thermo Fisher Cat # 23227) and final concentration with addition to 2x Laemmli buffer (4% SDS, 5% 2-mercaptoethanol, 20% glycerol, 0.02% bromophenol blue, 125 mM Tris) was adjusted so that equal quantities of protein could be loaded for the sets.

### SDS-PAGE and Western Blotting

Total of 66, 50, and 55 μg/well protein sample was loaded for 12, 24, and 48 h treated samples respectively. Protein samples were run in 4–20% gradient SDS polyacrylamide gel (Bio-Rad Cat # 4561096) and transferred onto a PVDF membrane (Bio-Rad Cat # 1620177) by the wet transfer method. The membranes were blocked in 5% non-fat milk in TBST (50 mMTris, 150 mM NaCl, 0.1% Tween 20) for 1 h at room temperature, washed thrice with TBST for 5 min each. The membranes were then incubated with primary antibody solution at 4°C with gentle shaking overnight. The membranes were washed thrice with TBST for 5 min each. HRP linked secondary antibody binding was performed at room temperature for 1 h or 4°C overnight. The membranes were then washed thrice with TBST for 5 min each. Detection was done using ECL kit (Bio-Rad Cat # 1705062) and chemiluminescence was detected on the Bio-Rad ChemiDoc XRS+ system. The following antibodies and dilutions were used; anti-actin 1:100 (DSHB Cat# JLA20, RRID:AB_528068), anti-mCherry 1:10 (DSHB Cat# DSHB-mCherry-3A11, RRID:AB_2617430), anti-GFP (Novus Cat# NB 600-308, RRID:AB_341929), HRP-Goat anti mouse 1:4000 (Cloud-Clone Corp. Cat # SAA544Mu19), HRP-Goat anti rabbit 1:4000 (Cloud-Clone Corp. Cat # SAA544Rb19), anti-mCherry 1:1000 (Thermo Fisher Scientific Cat# PA5-34974, RRID:AB_2552323), HRP-Goat anti rat 1:4000 (Cloud-Clone Corp. Cat # SAA544Ra09).

## Results

### Expression Analysis of GFP-Ref(2)P

Ref(2)P (SQSTM1/p62 in mammals) is one of the cargo receptors that binds polyubiquitylated substrates and aids in their recruitment into autophagosomes that are marked for degradation. In *Drosophila*, Ref(2)P was first characterized for its role in sigma rhabdovirus multiplication (Dezelee et al., 1989). Recently, it was shown that Ref(2)P is the *Drosophila* ortholog of mammalian p62, and the conserved PB1 and UBA domains are necessary for protein aggregate formation (Nezis et al., 2008). Genetic and pharmacological experiments in *Drosophila* demonstrate that reduction of autophagy activity leads to an accumulation of Ref(2)P-positive protein aggregates, suggesting that it can be used as a marker of autophagic activity (Nezis et al., 2008; Bartlett et al., 2011; Devorkin and Gorski, 2014).

**FIGURE 1:**
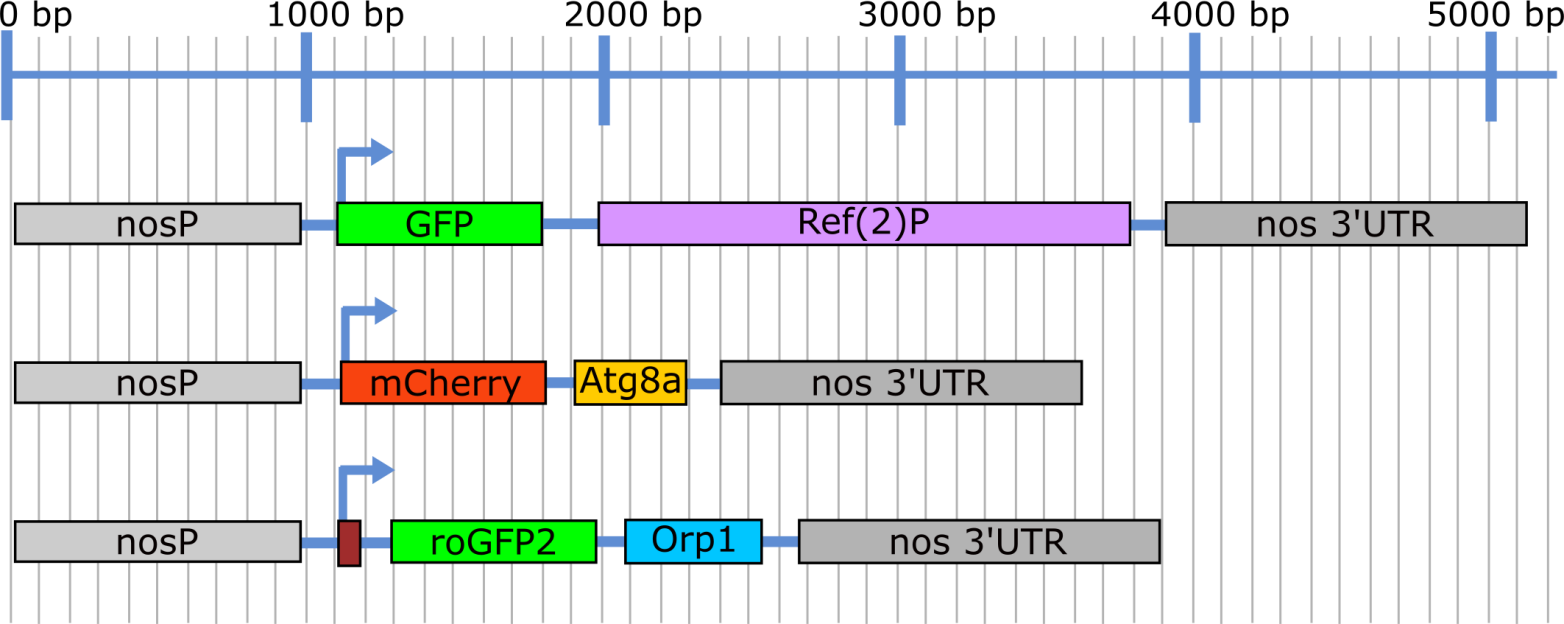
Illustration showing the expression cassettes for the three nosP transgenic constructs. Blue line depicts the size in base pairs (bp).

**FIGURE 2:**
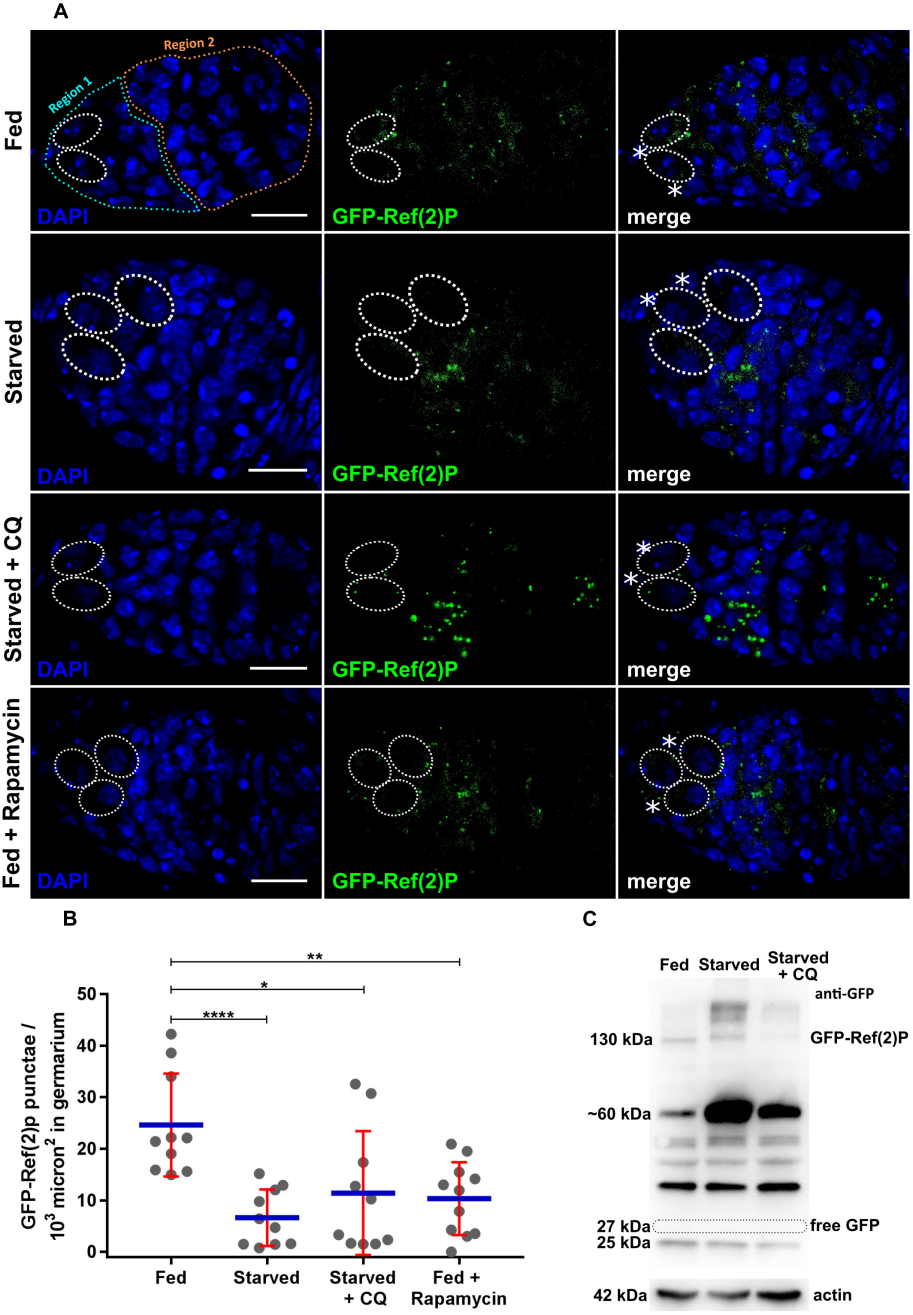
Expression of nosP-GFP-Ref(2)P nos 3′UTR. **(A)** GFP-Ref(2)P punctae in germarium of flies subjected to fed (for 6 days), fed + starved (2 days fed + 4 days), starved + Chloroquine (2 days fed + 4 days), fed + rapamycin (2 days fed + 4 days). Dotted ovals mark the GSCs and the asterisks mark the cap cells. Scale bar 10 μm. **(B)** Interleaved scatter graph showing distribution of GFP-Ref(2)P punctae in germaria as a function of various treatments carried out for 12 h. Error bars represent SD in red and the mean is blue. *n* = 10 for fed, starved, starved and chloroquine and 11 for fed with rapamycin respectively. ^∗^*p* < 0.05, ^∗∗^*p* < 0.01, ^∗∗∗∗^*p* < 0.0001. **(C)** Western blot analysis using anti-GFP antibodies of ovarian extracts expressing GFP-Ref(2)P. 130 kDa GFP-Ref(2)P band could be detected in fed, starved and starved + CQ treated ovaries for 48 h. Loading control actin is shown below for the same samples.

We created a pCasper4 based plasmid that possesses 935 bp *nanos* promoter and a 1236 bp 3′ UTR of *nanos* that stabilizes transcripts in the germline to generate GFP-Ref(2)P (Doren et al., 1998; Rørth, 1998; Nilangekar and Shravage, 2018; Figure 1). Nanos is a maternally expressed and is required for the process of oogenesis and egg production. Nanos expression is detected very early in primordial germ cells of the embryo which later become the germline cells of larval gonads and of the adult ovaries (Wang et al., 1994; Rørth, 1998; Dansereau and Lasko, 2008). We tested larval and adult ovaries for GFP-Ref(2)P expression and its subcellular localization. GFP-Ref(2)P puncta could be readily detected in the GSCs of both larval ovaries and adult ovaries (Figure 2 and Supplementary Figures S1A,B). Larval ovaries from these transgenic lines showed high levels of GFP-Ref(2)P in the developing GSCs. GFP-Ref(2)P expression was weaker and diffused in the support cells and in the region where niche cells would develop (Supplementary Figure S1A). Several of the germaria and late egg chambers possessed rod-shaped distribution of GFP-Ref(2)P which was described previously (Nezis, 2012; Supplementary Figure S1D). The distribution of GFP-Ref(2)P puncta within the germarium exhibited differences. Interestingly, majority of GFP-Ref(2)P puncta were found to be localized in the region 2 (2a and 2b) of germarium while region 1 appeared to have very few or no punctate GFP structures. In our analyses upto 30% germaria did not possess GFP-Ref(2)P punctae (Supplementary Figure S2D). Antibodies against GFP and p62 were used to validate the expression of GFP-Ref(2)P during oogenesis. Significant overlap between anti-GFP and GFP-Ref(2)P punctate structures was observed and further supported by high Pearson’s coefficient. Approximately 60% of the GFP-Ref(2)P puncta were positive for anti-p62 as indicated by Pearson’s correlation coefficient (Supplementary Figures S2A–C).

The transgenic line was subjected to nutrient stress and pharmacological treatments to validate their utility in various autophagy assays. Females expressing GFP-Ref(2)P were grown on sucrose only diet (nitrogen starvation), rapamycin (autophagy inducer) and 4-hydroxy-chloroquine (CQ) (autophagosome-lysosome fusion inhibitor; for details please refer to methods sections) for varying periods of time (Klionsky et al., 2016). The treatments were carried out for 6, 12, 24, 48, and 96 h. The ovary morphology changes in response to these stress stimuli are shown for each treatment in Supplementary Figure S1C. The number of GFP-Ref(2)P puncta decreased significantly in germaria obtained from starved females as compared to females reared on nutrient-rich food indicating induction of autophagy. CQ neutralizes lysosomal pH which subsequently prevents autophagosome-lysosome fusion (Ahlberg et al., 1985; Yoon et al., 2010). Upon CQ treatment, the number of GFP-Ref(2)P puncta significantly increased, as compared to germaria obtained from starved females indicating a disruption autophagic degradation of Ref2P (Figure 2A). Rapamycin induces autophagy by inhibiting mTOR kinase (Noda and Ohsumi, 1998; Klionsky et al., 2016). Females grown on food containing rapamycin exhibited a significant reduction of GFP-Ref(2)P puncta suggesting a robust induction of autophagy in germaria (Figure 2A,B).

Autophagy dependent cleavage of GFP-Ref(2)P is one of the ways to monitor its degradation. GFP is comparatively resistant to lysosomal hydrolases due to its compact globular structure. The liberation of free GFP from GFP-Ref(2)P following its delivery to the lysosome and can be reliably assayed using an immunoblot assay (Pircs et al., 2012; Devorkin and Gorski, 2014; Klionsky et al., 2016). We subjected GFP-Ref(2)P transgenic females to starvation induced autophagy and CQ treatment, extracted protein from the ovaries and tested if free GFP is liberated (Figure 2C and Supplementary Figure S8). As seen from Figure 2C, the anti-GFP antibody could detect changes in the levels of GFP-Ref(2)P fusion protein (∼130 kDa) in fed, starved and CQ treated conditions. For instance, high molecular weight bands > 130 kDa [GFP-Ref(2)P aggregates] were detected in starved and CQ treated conditions. Interestingly, a number of bands ranging from ∼ 60kDa–25kDa, intermediate degradation products of GFP-Ref(2)P, could be detected with the anti-GFP antibody. In particular, the predominant GFP-Ref(2)P intermediate degradation product was detected at ∼60 kDa. This GFP-Ref(2)P intermediate degradation product was seen to be enriched in starved condition and its abundance was found to be decreased upon CQ treatment. Free GFP (∼27 kDa) was not detected in any of the conditions tested and possible reasons are discussed in later section (Pircs et al., 2012; Figure 2C and Supplementary Figures S8A,B). Taken together, our data suggest that the GFP-Ref(2)P transgenic lines could be used as a reporter of autophagy in the female germline of *Drosophila*.

### Expression Analysis of mCherry-Atg8a

Due to its ubiquitous expression in most tissues, Atg8a has been routinely used to monitor autophagy using various techniques including western blotting and immunofluorescence microscopy. Atg8a has been shown to be induced in germline cells as well as follicle cells in response to starvation during *Drosophila* oogenesis (Nezis et al., 2010a; Barth et al., 2011; Hegedus et al., 2016; Bali and Shravage, 2017).

**FIGURE 3:**
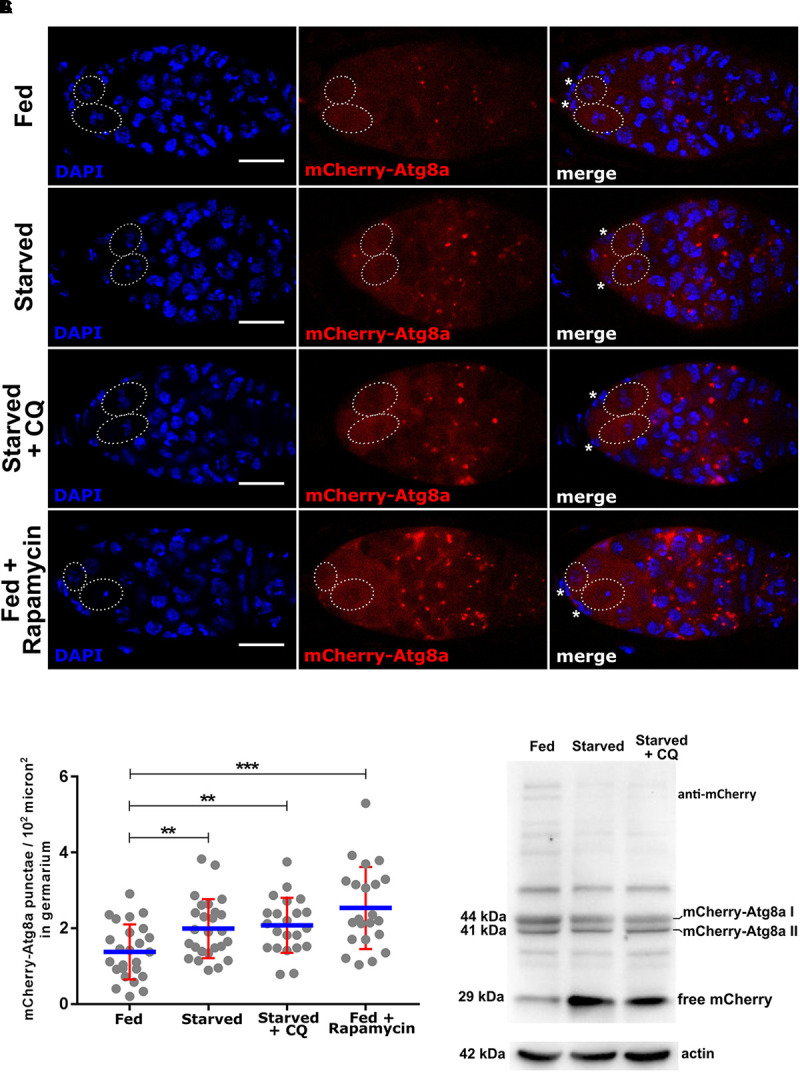
Expression of nosP mCherry-Atg8a nos 3′UTR. **(A)** mCherry-Atg8a punctae in germarium of flies subjected to fed, starved, starved and chloroquine or fed with rapamycin treatment. Dotted ovals mark the GSCs and the asterisks mark the cap cells. Scale bar 10 μm. **(B)** Interleaved scatter graph showing distribution of mCherry-Atg8a punctae in germarium of flies subjected to fed (for 6 days), fed + starved (2 days fed + 4 days), starved + Chloroquine (2 days fed + 4 days), fed + rapamycin (2 days fed + 4 days). Error bars represent SD in red and the mean is blue. *n* = 25, 26, 22 and 22 for fed, starved, starved and chloroquine and fed with rapamycin respectively. ^∗∗^*p* < 0.01, ^∗∗∗^*p* < 0.001. **(C)** Western blot analysis using anti-mCherry antibodies of ovarian extracts expressing mCherry-Atg8a. 44, 41, and 29 kDa bands corresponding to mCherry-Atg8a-I, mCherry-Atg8a-II, and free mCherry respectively could be detected in fed, starved and starved + CQ treated ovaries for 48 h. Loading control actin is shown below for the same samples.

Transgenic lines expressing *mCherry-Atg8a* under the *nanos* promoter were generated as described in materials and methods. mCherry-Atga8a expression was monitored in larval ovaries. mCherry-Atg8a was found to be diffused in larval GSCs but was punctate in the differentiated cells of larval ovaries. Unlike in GFP-Ref(2)P transgenic lines, no signal was detected in the fat body cells surrounding the larval ovaries (Supplementary Figure S3A). mCherry-Atg8a puncta could be detected in germaria and late egg chambers in adult ovaries (Figure 3A and Supplementary Figures S3B, 4A).

The most dramatic change in localization of Atg8a appears when autophagy is induced, where cytoplasmic Atg8a in fed conditions localizes to autophagosomes and autolysosomes and is observed as punctate structures in fluorescence microscopy. Ovaries from adult females reared on 20% sucrose (nitrogen deprivation for 1–4 days) and fed flies (yeast) were dissected and assayed for mCherry-Atg8a expression (Supplementary Figure S3C). Our analyses suggested upregulation of autophagy by 24 h of starvation and a significant upregulation of autophagy by 48 h of starvation (Supplementary Figure S3D). However, we could detect an increase in total mCherry-(Atg8a) intensity in the GSCs in starved vs. fed GSCs (Supplementary Figure S4E). A significant increase in the number of mCherry-Atg8a punctae was detected in the entire germaria of ovaries dissected from fed vs. starved females (Figure 3A,B). The increase in mCherry-Atg8a punctae was concentrated in region 2 of germarium (Figure 3A). We next tested if disrupting autophagosome-lysosome fusion led to the accumulation of mCherry-Atg8a puncta. As expected, mCherry-Atg8a positive punctate structures accumulated within the germaria isolated from females grown on food containing CQ. In addition, germaria from rapamycin-treated transgenic females exhibited significantly higher number of mCherry-Atg8a puncta (autophagosomes and autolysosomes) as compared to transgenic females reared on nutrient rich food (Figure 3A,B). These data suggest that nosP-mCherry-Atg8a transgenic line could be used to monitor autophagy in the germarium.

Immunoblot analyses of mCherry-Atg8a (Atg8a) is recommended as it provides independent confirmation of autophagy induction (Mauvezin et al., 2014; Mulakkal et al., 2014; Nagy et al., 2015; Klionsky et al., 2016; Lörincz et al., 2017). The degradation of mCherry-Atg8a II fusion protein within the lysosome is differential. The Atg8a-II part of the fusion protein is degraded rapidly while the mCherry part is relatively resistant to destruction by the lysosomal enzymes. The liberation of free mCherry can be reliably assayed using immunoblot analysis to infer autophagic flux (Mauvezin et al., 2014; Nagy et al., 2015; Klionsky et al., 2016). Transgenic females expressing mCherry-Atg8a were subjected to nutrient limitation and CQ treatment and, the protein from the ovaries was assayed for mCherry liberation using an immunoblot assay. In fed, starved and CQ treatment, both mCherry-Atg8a-I (∼44 kDa) and mCherry-Atg8a-II (∼41 kDa) forms were detected. As expected there was an increase in the formation of mCherry-Atg8a-II and corresponding increase in liberation of free mCherry (∼29 kDa) in starved conditions. As compared to fed and starved conditions, in CQ treated animals, the mCherry-Atg8a-II form was detected at significantly higher levels, however, the liberation of free mCherry was inhibited indicating impaired lysosomal destruction of mCherry-Atg8a (Figure 3C and Supplementary Figures S8C,D). These data suggest that the mCherry-Atg8a transgenic lines could be used to monitor autophagy during oogenesis.

We tested if mCherry positive punctate structures expressed during oogenesis from the transgene are positive for Atg8a. To test this, anti-GABARAP antibodies which also detect *Drosophila* Atg8a were utilized (Lörincz et al., 2017; Tusco et al., 2017). mCherry positive puncta colocalized with anti-GABARAP positive puncta both in the germarium and late stage egg chamber confirming expression of mCherry-Atg8a fusion protein (Supplementary Figure S4A).

UASp-mCherry-Atg8a autophagy reporter has been previously described and has been demonstrated to monitor autophagy in the nurse cells during oogenesis (Jacomin and Nezis, 2016). In this study, we compared UASp-mCherry-Atg8a (nanos-Gal4VP16 X UASp-mCherry-Atg8a) and nosP-mCherry-Atg8a simultaneously in starvation-induced autophagy assay in the ovaries. mCherry-Atg8a punctae are not significantly different between germaria dissected from nanos-Gal4 driven UASp-mCherry-Atg8a females and nosP-mCherry-Atg8a females (Supplementary Figures S4D,E). nanos-Gal4VP16 X UASp-mCherry-Atg8a germaria possess higher levels of cytoplasmic mCherry-Atg8a in region 2 (4, 8 and 16 cell cysts) of germaria. Upon nutrient limitation, the increase in the number and fluorescence intensity of mCherry-Atg8a punctae in both nanos-Gal4 X UASp-mCherry-Atg8a expressing germaria and nanosP-mCherry-Atg8a germaria is comparable (Supplementary Figures S4D,E).

We checked if nosP-mCherry-Atg8a transgene can rescue lethality associated with *Atg8a^KG07569^* transposon insertion. Homozygous *Atg8a^KG07569^* insertion mutants lack detectable levels of Atg8a protein as tested by western blotting technique (Chang et al., 2013). The rescue experiment is designed to test if the expression level of mCherry-Atg8a from nanos promoter is adequate enough for the complementing the deficiency of Atg8a in homozygous *Atg8a^KG07569^* mutant. Two separate nosP-mCherry-Atg8a insertions were tested for rescue of lethality of *Atg8a^KG07569^* mutant. Our data indicate that both transgenes were capable of complementing the deficiency of Atg8a in homozygous *Atg8a^KG07569^* mutant. Mendelian ratios of inheritance were observed (Supplementary Figure S5B). Taken together, these data suggest that nosP-mCherry-Atg8a transgenic line could be used to monitor autophagy in GSCs, germaria and nurse cells during oogenesis.

### Measurement of Autophagy Flux

**FIGURE 4:**
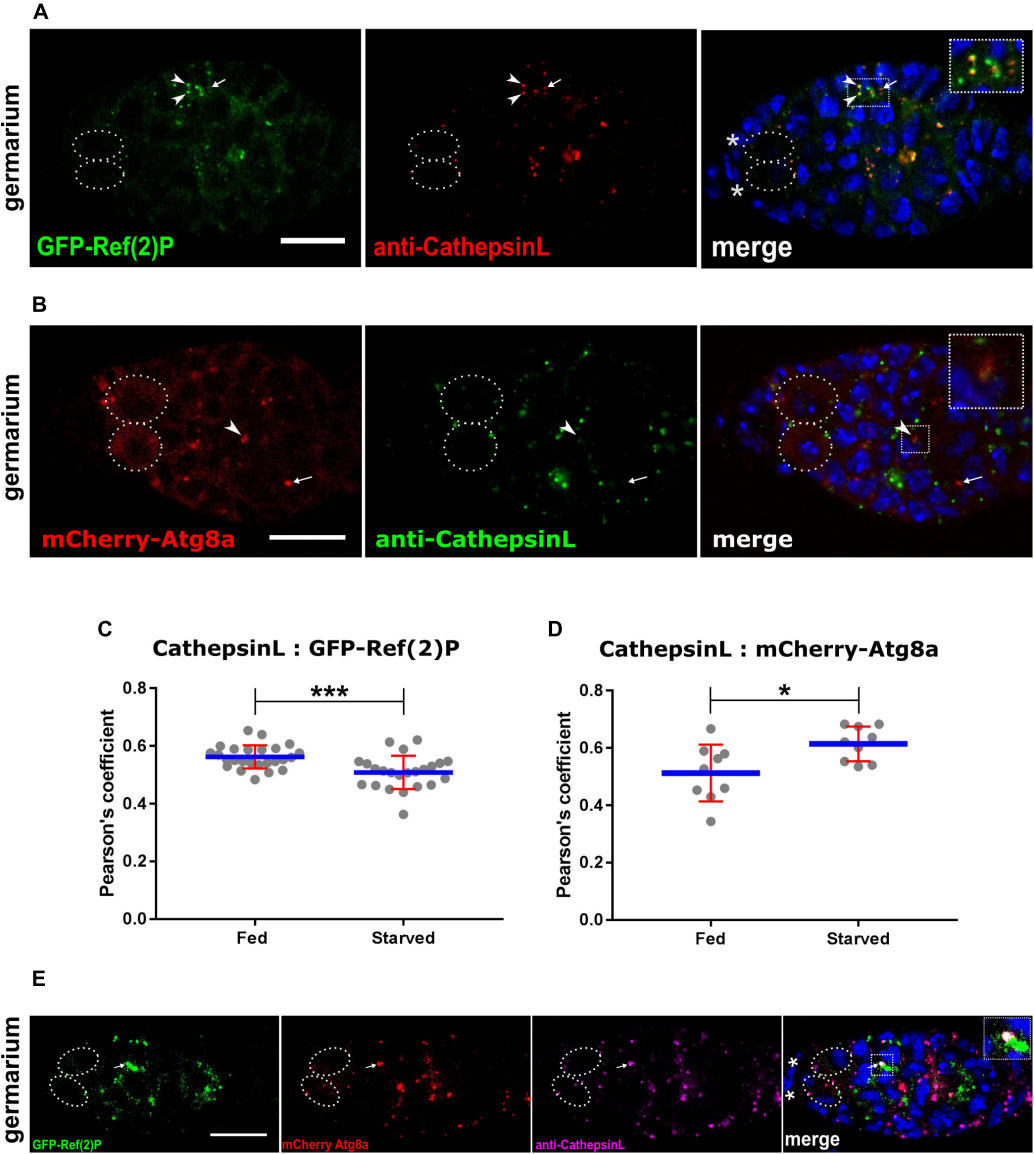
Measurement of autophagy flux together with CathepsinL. **(A)** GFP-Ref(2)P punctae colocalizing with CathepsinL region (lysosomes). Arrowheads point to colocalized punctae and arrows indicate Cathepsin-L alone punctae. Inset shows enlarged region of Ref(2)P sequestered by lysosomes. Dotted ovals mark the GSCs and the asterisks mark the cap cells. Scale bar 10 μm. **(B)** mCherry-Atg8a punctae colocalizing with CathepsinL region (lysosomes). Arrowheads point to colocalized punctae (autolysosomes) and arrows indicate mCherry-Atg8a alone punctae (autophagosomes). Inset shows enlarged region with an autolysosome. Dotted ovals mark the GSCs and the asterisks mark the cap cells. Scale bar 10 μm. **(C)** Starvation induced autophagic degradation of Ref(2)P. Interleaved scatter graph showing Pearson’s coefficient as measure of colocalization of Cathepsin-L and GFP-Ref(2)P in germarium of fed and starved nosP-GFP-Ref(2)P transgenic flies immunostained with anti-Cathepsin-L antibody. Error bars represent SD in red and the mean is blue. *n* = 25 for fed (6 days) and *n* = 23 for starved (2 days fed + 4 days starved). ^∗∗∗^*p* < 0.001. **(D)** Induction of autophagy upon starvation. Interleaved scatter graph showing Pearson’s coefficient as measure of colocalization (autolysosome) of Cathepsin-L (lysosome) and mCherry-Atg8a (autophagosomes) in germarium of fed and starved nosP-mCherry-Atg8a transgenic flies immunostained with anti-Cathepsin-L antibody. Error bars represent SD in red and the mean is blue. *n* = 9 for both fed and starved. ^∗^*p* < 0.05. **(E)** Ref(2)P sequestered by autophagosomes and fused to lysosomes. Germarium of mCherry-Atg8a; GFP-Ref(2)P flies immunostained for Cathepsin-L. Inset shows enlarged region having a puncta (arrow) positive for GFP, mCherry as well as Cathepsin-L. Dotted ovals mark the GSCs and the asterisks mark the cap cells. Scale bar 10 μm.

Autophagic flux is a measure of degradation of autophagic cargo within the lysosomes. There are several methods to measure autophagic flux. Atg8a based assays measure autophagic carrier flux and not autophagic cargo/substrate flux *per se* (Tanida et al., 2005; Klionsky et al., 2016). While Ref(2)P based assays are considered to be a better measure of autophagic flux. This is due to the fact that Ref(2)P has ability to bind to polyubiquitinated proteins/substrates which allows for their delivery to the autophagosome. In addition, upon starvation and rapamycin treatment, Ref(2)P displays the largest degree of change which can be quantified reliably (Klionsky et al., 2016). To test if transgenic lines expressing GFP-Ref(2)P and mCherry-Atg8a individually could be used for assaying flux we stained them for CathepsinL. CathepsinL is cysteine protease that is a component of the lysosomal acid proteases and used as a lysosomal marker (Klionsky et al., 2016). GFP-Ref(2)P (green) puncta localized close to CathepsinL (red) dots in the germarium and nurse cell cytoplasm in late egg chambers (Figure 4A and Supplementary Figure S6A). As expected three different types of puncta were visible viz. green puncta (GFP-Ref(2)P aggregates alone or vesicle-bound), and red puncta (lysosomes) could be detected along with yellow puncta (autolysosomes). Pearson’s coefficient of GFP-Ref(2)P and CathepsinL colocalization was found to be significantly lower in starved conditions as compared to fed conditions (Figure 4A,C). In contrast, mCherry-Atg8a (red) puncta colocalized with CathepsinL (green dots) stained lysosomes. In this experiment, three different puncta were visible: red puncta which represent autophagosomes, yellow puncta which correspond to autolysosomes, and green dots that depict lysosomes. Pearson’s coefficient showed a significant increase of mCherry-Atg8a and CathepsinL colocalization in starved vs. fed conditions (Figure 4B,D and Supplementary Figure S6B).

To get a better estimate of autophagic flux transgenic lines expressing both GFP-Ref(2)P and mCherry-Ag8a were crossed together and ovaries were dissected and stained for CathepsinL. Several puncta positive for GFP-Ref(2)P, mCherry-Atg8a and CathepsinL were visible in fed germaria (Figure 4E and Supplementary Figure S6C). Taken together, our analyses suggest that these transgenic lines could be used to measure changes in autophagic flux in combination with CathepsinL.

### The Utility of Transgenes in Genetic Screens

**FIGURE 5:**
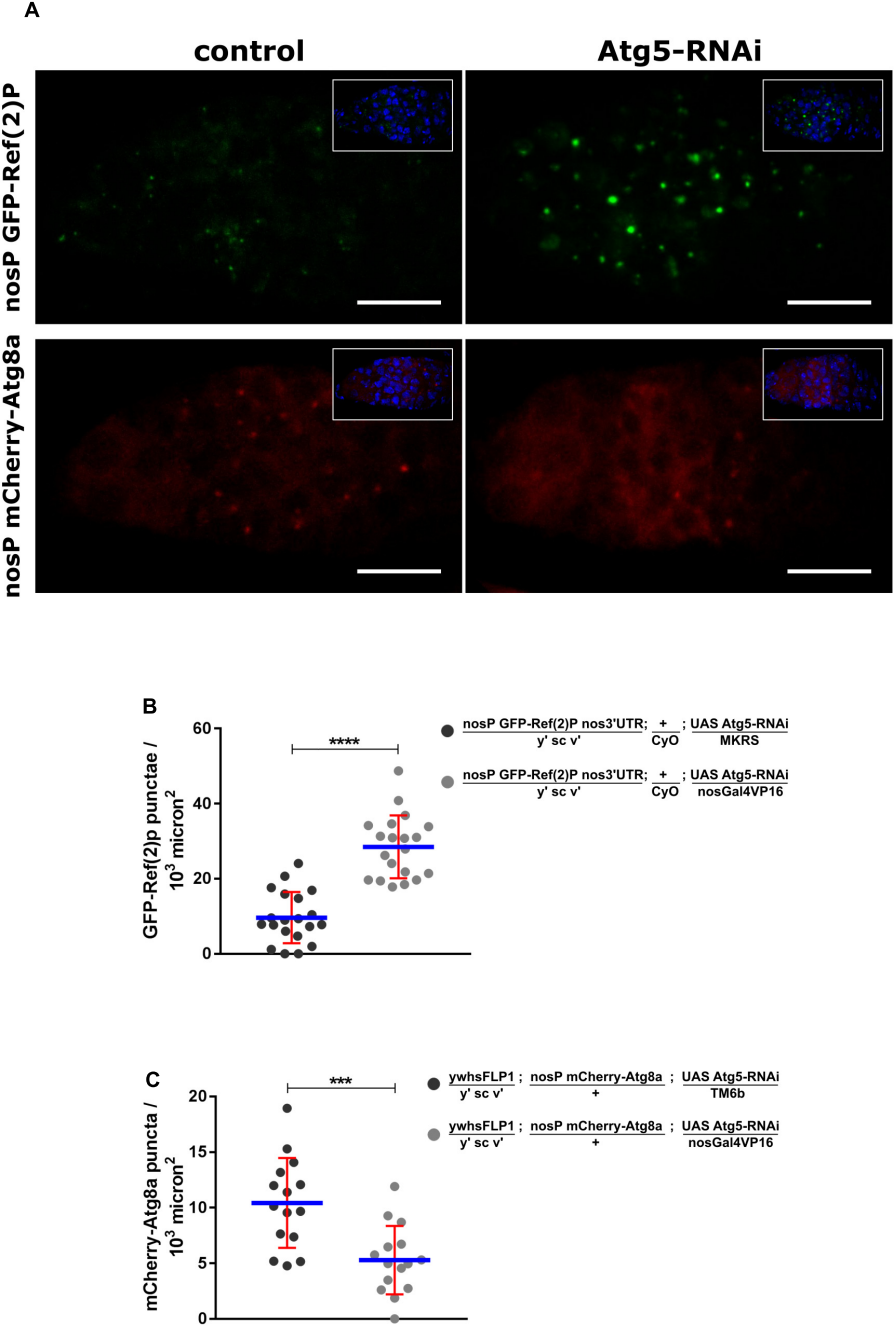
Utility of transgenic reporter lines in RNAi based genetic screen. **(A)** Germaria of nosP GFP-Ref(2)P nos 3′UTR and nosP mCherry-Atg8a nos 3′UTR transgenic flies in background of either no knockdown (control) or knockdown of Atg5 by RNAi in the germline. Scale bar 10 μm. **(B)** Interleaved scatter graph showing the increase in GFP-Ref(2)P punctae upon Atg5 knockdown. Error bars represent SD in red and the mean is blue. *n* = 20 for both the data sets. ^∗∗∗∗^*p* < 0.0001. **(C)** Interleaved scatter graph showing the decrease in mCherry-Atg8a punctae upon Atg5 knockdown. Error bars represent SD in red and the mean is blue. *n* = 15 for both the data sets. ^∗∗∗^*p* < 0.001.

One of the advantages of the *Drosophila* model system is the ability to conduct rapid forward and reverse genetic screens (St Johnston, 2002). We examined whether the GFP-Ref(2)P and mCherry-Atg8a reporters could be of utility in genetic screens. A double-stranded inverse-repeat (IR) construct designed to target and knockdown Atg5 (Atg5IR) was expressed specifically in the germline cells using nanos-Gal4VP16 (Doren et al., 1998; Ni et al., 2008). The expression and cytoplasmic localization of GFP-Ref(2)P and mCherry-Atg8a was monitored in Atg5 knockdown cells. Indeed, as compared to control, germaria expressing Atg5IR failed to degrade GFP-Ref(2)P as seen from the accumulation of GFP positive punctate structures. Moreover, these GFP-Ref(2)P puncta appeared to be larger (GFP-Ref(2)P aggregates) as compared to GFP-Ref(2)P in control germaria indicating impaired autophagy (Figure 5A,B). Knockdown of Atg5 in germaria led to the disruption of punctate localization of mCherry-Atg8a indicating reduced autophagosome formation when compared to control germaria. mCherry-Atg8a was predominantly cytoplasmic in Atg5IR expressing germaria further supporting a decrease in autophagosome formation (Figure 5A,C). Similar results were obtained when Atg8aIR was expressed in the germ cells (Supplementary Figure S5A). Taken together, these data suggest that both GFP-Ref(2)P and mCherry-Atg8a could be used in conducting genetic screens to identify modules contributing to the maintenance and induction of autophagy.

### Expression Analysis of mito-roGFP2-Orp1

Mitochondria are susceptible to oxidative damage due to the production of mitochondrial reactive oxygen species (mROS) which include OH^-^, O_2_^-^ and H_2_O_2_ (Dröge, 2002; Dan Dunn et al., 2015). Damaged mitochondria lose their redox potential and are subsequently cleared via mitophagy. mROS are products of normal as well as altered cell physiology and can provide valuable information of cellular health (Dröge, 2002; Shadel and Horvath, 2015; Tan et al., 2017). We generated a germline mitochondria-specific sensor for H_2_O_2,_ termed here as mito-roGFP2-Orp1.

**FIGURE 6:**
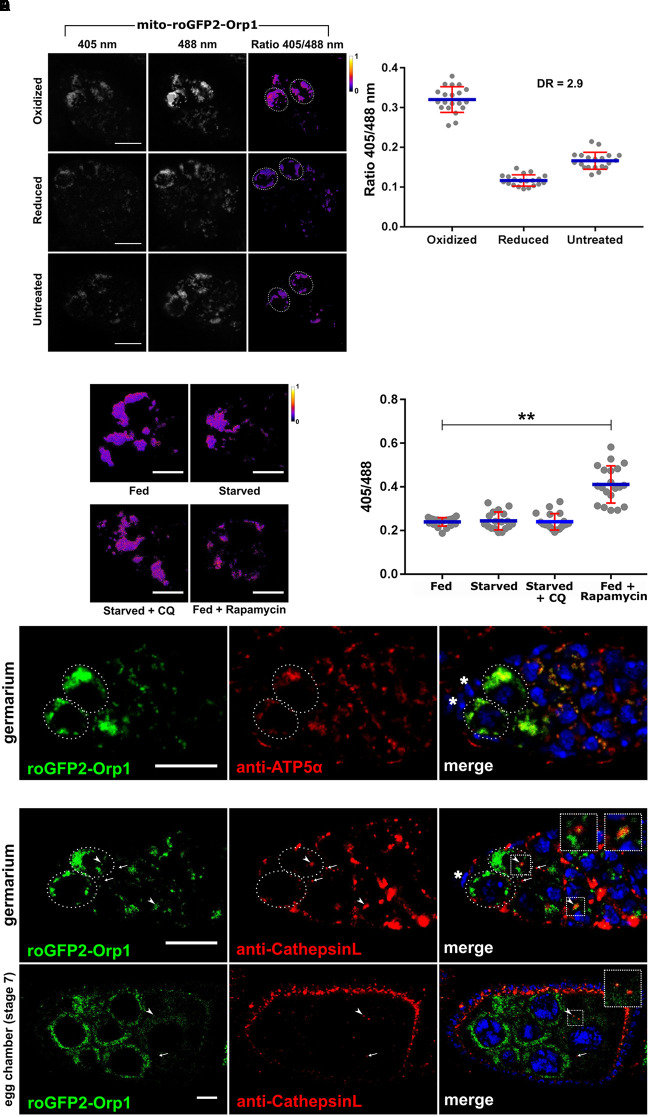
Expression and function of nosP-mito-roGFP2-Orp1-nos 3′UTR reporter. **(A)** mito-roGFP2-Orp1 monitors the redox state in the GSCs. Ratio of emission at 405 and 488 nm obtained as a response of the reporter to applied oxidant (DA) or reductant (DTT). Dotted ovals mark the GSCs. Scale bar 10 μm. **(B)** Interleaved scatter graph showing ratiometric shift of excitation. Error bars represent SD in red and the mean is blue. *n* = 20 for each condition. DR stands for dynamic range. **(C)** Redox state of GSCs of mito-roGFP2-Orp1 transgenic flies for fed (for 6 days), fed + starved (2 days fed + 4 days), starved + Chloroquine (2 days fed + 4 days), fed + rapamycin (2 days fed + 4 days) treatment. Scale bar 5 μm. **(D)** Interleaved scatter graph showing ratiometric shift of excitation fed (for 6 days), fed + starved (2 days fed + 4 days), starved + Chloroquine (2 days fed + 4 days), fed + rapamycin (2 days fed + 4 days) treatment. Error bars represent SD in red and the mean is blue. *n* = 20, 18, 19, and 21 for fed, fed + starved, starved + Chloroquine, fed + rapamycin respectively. ^∗∗^*p* < 0.01. **(E)** Germarium of mito-roGFP2-Orp1 transgenic flies immunostained with anti-ATP5α antibody that marks the mitochondria. Dotted ovals mark the GSCs and the asterisks mark the cap cells. Scale bar 10 μm. **(F)** mito-roGFP2-Orp1 (green) in the germarium and a stage 7 egg chamber shows co-localization with lysosomes marked by Cathepsin-L (red). Some lysosomes are GFP positive (yellow) marked by arrow heads while the arrows mark GFP negative lysosomes. Inset shows the enlarged region of the fusion of mitochondrion (presumably in the autophagosome) and lysosome. Dotted ovals mark the GSCs and the asterisk marks the cap cell. Scale bar 10 μm.

The expression of this H_2_O_2_ sensor was tested in the larval and adult ovaries. mito-roGFP2-Orp1 was detected in the entire germarium with very strong expression in the GSCs (Tan et al., 2017). mito-roGFP2-Orp1 distribution in GSCs appeared to be predominantly at the periphery of the GSC nucleus with random distribution in cysts present in region 2 of the germarium. In late stage egg chambers, mito-roGFP2-Orp1 was also detected surrounding the nurse cell nucleus. The follicle cells were devoid of any GFP expression (Figure 6A,F). mito-roGFP2-Orp1 can be detected in the larval ovaries with predominant expression within the developing GSCs. GFP signal was not detected in differentiating cells of the larval ovaries or in the fat tissue surrounding the larval ovaries (Supplementary Figure S7).

We tested if mito-roGFP2-Orp1 could be used to sense the mitochondrial redox potential within the germarium and GSCs as the mito-roGFP2-Orp1 cassette carries a mitochondrial localization signal at the N-terminal. This reporter senses H_2_O_2_ first via Orp1 which oxidizes when exposed to H_2_O_2_ and relays the oxidation state to roGFP2 which leads to shift in its excitation maxima of roGFP2 from 488 to 405 nm (Albrecht et al., 2011, 2014). The ovarian tissue was subjected to complete oxidation and reduction using chemical agents, and the excitation maxima of the reporter was tested in the GSCs. As shown in Figure 6A,B this redox sensor has the ability to sense both reduced and oxidation states of mitochondria within the GSCs. The dynamic range of the reporter that measure the maximum oxidation and maximum reduction and was found to be 2.9 in the GSCs (Figure 6B).

To determine if mito-roGFP2-Orp1 expression or its distribution changes in response to different stress stimuli, we subjected mito-roGFP2-Orp1 expressing females to nutrient limitation, CQ treatment and rapamycin exposure. As compared to nutrient-rich conditions, starvation of the mito-roGFP2-Orp1 expressing females led to reduced roGFP2 expression within the GSCs indicating turnover of mitochondria. In contrast, exposure to CQ during nutrient limitation restored the roGFP2 expression in the GSCs indicating disruption of mitophagy. Rapamycin treated germaria exhibited reduced roGFP2 expression indicating upregulation of mitophagy leading to clearance of mitochondria within the GSCs (Figure 6C). We further examined the shift in excitation maxima of roGFP2 from 488 to 405 nm in response to these stress stimuli. A shift in excitation maxima of roGFP2 was not detected when females expressing the transgene were grown on nutrient-rich, nutrient-deprived or in presence of CQ. However, we observed a significant shift in excitation maxima of roGFP2 from 488 to 405 nm indicating increased oxidative stress in GSCs upon rapamycin exposure (Figure 6D).

We confirmed that the mito-roGFP2-Orp1 expression is indeed mitochondrial by co-staining with an anti-ATP5α antibody. Our data suggest near complete overlap of the roGFP2 signal with anti-ATP5α suggesting mitochondrial-specific expression of the mito-roGFP2-Orp1 transgene (Figure 6E). To test if mito-roGFP2-Orp1 could be used to track mitophagy we stained ovaries dissected from flies expressing mito-roGFP2-Orp1 with CathepsinL. As seen in Figure 6F we could detect colocalization of GFP (green mitochondria) and CathepsinL (red lysosomes) in germarium and stage 7 egg chambers suggesting occurrence of mitophagy.

## Discussion

Here we describe generation and characterization of three transgenic lines that aid in assaying for autophagy, mitochondrial H_2_O_2_ production and mitophagy specifically in the GSCs. The purpose of generating these lines was multifold. First, to avoid problems linked with Gal4/UASp mediated overexpression of GFP-Ref(2)P, mCherry-Atg8a and mito-roGFP2-Orp1. Second, to validate expression and localization of GFP-Ref(2)P, mCherry-Atg8a and assay for H_2_O_2_ production in the GSC and their progeny. Third, to document any differences in expression in different cell types within the germarium including GSCs. And finally, to test the utility of the transgenes in the measurement of autophagy flux and mitophagy.

Although Gal4/UASp is a powerful tool to express genes of interest in the germline cells, it has limitations similar to those documented in the case of GAL4/UAS. Gal4/UASp driven transgenes have mosaic/patchy expression within the tissue (Brand and Perrimon, 1993). This leads to significant differences in the expression levels of the transgenes and in addition the variance is dependent on cell type within the tissue (Duffy, 2002). Our analyses with nanosGal4/UASp-mCherry-Atg8a in the germarium confirms the patchy mCherry-Atg8a expression (Supplementary Figure S4B, fed panel vs. germarium panel). Since Gal4/UASp too is a binary system the utility of this system in genetic screens is reduced as the transgene is expressed only in the F1 generation. This can be disadvantageous as a potential positive hit identified from a genetic screen needs additional experimentation in F2 generation for confirmation. Further, GAL4/UASp driven transgenes may lead to over-production of the protein leading to erroneous results and complicate image analyses (Supplementary Figure S4B, note the excess deposition of mCherry-Atga in the developing cysts). The nanos promoter driven transgenes provide a superior alternative to Gal4/UASp driven transgenes, as the transgenes are expressed directly from nanos promoter. This leads to production of moderate to low levels of mRNA and protein within the germline cells and it is possible to conduct F1 genetic screens (Figure 2, 3, 6).

The earliest expression of GFP-Ref(2)P in the larval ovaries was detected in the GSCs (Supplementary Figure S1). Fat body cells surrounding the larval ovaries also exhibited detectable levels of GFP-Ref(2)P expression. This was unexpected as *nanos* promoter is specific to germ cells and it has not been reported to be active in fat cells (Rørth, 1998; Gelbart and Emmert, 2013). However, we cannot rule out prolonged persistence of GFP-Ref(2)P deposited in the egg through larval development. *Drosophila* Ref(2)P was first characterized for its role in sigma rhabdovirus multiplication. Ref(2)P is mammalian homolog of p62, and impaired autophagy causes an accumulation of Ref(2)P- aggregates, indicating that it can be used as a marker of autophagic activity (Nezis et al., 2008, 2010b; Bartlett et al., 2011). Nezis et al. (2008) have also showed Ref(2)P expression in the nurse cells as well as in the follicle cells during oogenesis. Our data show that GFP-Ref(2)P is expressed in the GSCs and nurse cells in late egg chambers with punctate distribution in fed conditions (Devorkin and Gorski, 2014; Jacomin and Nezis, 2016). It is worth noting that a certain percentage of germaria analyzed did not bear any GFP-Ref(2)P puncta. Interestingly, in few germaria and nurse cells from late stage egg chambers, Ref(2)P was found to be localized in rod shaped form described in Nezis (2012). We also report occurrence of these rod-shaped structures in the germarium (Supplementary Figure S1D). Further, GFP-Ref(2)P puncta were found to be concentrated in region 2a and 2b of germarium. This region-specific localization was also observed in germaria from starved flies but was significantly lower. These results support earlier reports that GFP-Ref(2)P in combination with immunoblot assays can be used to measure autophagic flux in *Drosophila* ovaries (Pircs et al., 2012; Devorkin and Gorski, 2014; Mauvezin et al., 2014; Lörincz et al., 2017).

GFP-Ref(2)P levels are maintained at steady state in fed conditions (Pircs et al., 2012). Upon starvation, in addition to the expected band of GFP-Ref(2)P at 130kd, high molecular weight bands were also observed indicating formation of GFP-Ref(2)P aggregates (Figure 2C and Supplementary Figures S8A,B). This also corelated with increased turnover of GFP-Ref(2)P as GFP-Ref(2)P intermediates could be detected at 12, 24, and 48 h of starvation (Figure 2 and Supplementary Figure S8). ∼ 60 kDa GFP-Ref(2)P intermediate species was seen to be predominant form at 48 h of starvation and its abundance decreased upon CQ treatment indicating inhibition of lysosomal activity. This intermediate form of GFP-Ref(2)P was also reported by Juhasz group (Pircs et al., 2012). Surprisingly, free GFP was not detected at 12, 24, and 48 h of nutrient-limitation and CQ treatment. This could be due to altered configuration of GFP due to exposure to acidic pH that does not allow for binding of the antibodies in the polyclonal serum. Alternatively, this could be due to a reduction in the autophagy carrier flux or due to efficient turnover of the GFP protein. Additionally, the possibility of degradation of a fraction of GFP-Ref2P pool through the proteosomal pathway cannot be ruled out (Pircs et al., 2012; Devorkin and Gorski, 2014; Klionsky et al., 2016).

Both mCherry-Atg8a-I and mCherry-Atg8a-II were detected in nutrient-rich conditions indicating basal autophagic activity during oogenesis. Correspondingly, low levels of free mCherry were detected in fed conditions as seen with the appearance of ∼29 kDa band (free mCherry). In starved ovaries, along with mCherry-Atg8a-I and mCherry-Atg8a-II forms, increased levels of free mCherry could be detected indicative of upregulation of autophagy and increase turnover of mCherry-Atg8a within the autolysosomes. These changes could be detected as early as 12 h post nutrient-deprivation. Upon CQ treatment, mCherry-Atg8a-II form was found to accumulate as compared to starved condition, with a corresponding decrease in the formation of free mCherry indicating impaired degradation of mCherry-Atg8a within the autolysosomes. These data indicate that the ovarian tissue responds to nutrient and pharmacological stress by upregulating or downregulating autophagy as expected (Figure 3 and Supplementary Figure S8; Barth et al., 2011).

We also detected CathepsinL in germaria expressing GFP-Ref(2)P and estimated the colocalization status of the two proteins. Our data suggest a significant overlap between GFP-Ref(2)P and CathepsinL indicating that Ref(2)P positive protein aggregates are indeed targeted to the lysosomes. Also, the measure of overlap decreases in germarium of starved flies suggesting increased degradation/turnover of Ref(2)P.

Previous studies have established transgenes of Atg8a that express in the germline. The UASp-mCherry-Atg8a transgene can be expressed only in presence of a germline driver (nosGal4VP16) and may lead to complications in interpretation of autophagy due to overloading of the cells with mCherry-Atg8a protein (Supplementary Figure S4; Nezis et al., 2009; Jacomin and Nezis, 2016). We have previously generated and characterized a mCherry-Atg8a fusion transgene which expresses under the endogenous promoter of Atg8a as part of investigating regulatory genetic elements of autophagy genes (Bali and Shravage, 2017). However, the expression levels of mCherry-Atg8a from the endogenous promoter appeared to have weak expression in the germline (unpublished). The mCherry-Atg8a transgenic line presented in this study has a strong expression in the germline cells and their progeny. As reported previously mCherry-Atg8a is predominantly found in region 2a and 2b of the germarium (Nezis et al., 2009). mCherry-Atg8a could also be detected in GSCs and hence could be used to monitor autophagy in the GSCs (Barth et al., 2011; Zhao et al., 2018). Upon starvation, mCherry-Atg8a puncta were found to be abundant in region 2a and 2b of germarium. Interestingly, we did not detect any increased mCherry-Atg8a puncta in GSCs upon starvation suggesting that GSC may be protected from starvation-induced autophagy. However, mCherry-Atg8a intensity appeared to be elevated in starved GSCs vs. Fed GSCs, where Atg8a may be participating in non-autophagic functions (Subramani and Malhotra, 2013). Additional experiments need to be performed to get better insights into this interesting phenomenon. mCherry-Atg8a puncta accumulated in germaria upon nutrient deprivation and in response to CQ treatment. Further rapamycin treatment too activated autophagy leading to increase in mCherry-Atg8a puncta. Rapamycin treatment stimulated the strongest response in germaria. These data suggest that nosP-mCherry-Atg8a transgene could be used to monitor basal as well as stress induced autophagy during oogenesis. Increased colocalization of mCherry-Atg8a and CathepsinL in starved germaria support the utility of this transgene for quantifying autophagy flux. Taken together, we believe that these germline specific Ref(2)P and Atg8a reporter will aid in measurement of autophagy flux during gametogenesis and under different conditions of stress. It will also allow measuring autophagy during early embryogenesis as these proteins are deposited in the egg (Forrest and Gavis, 2003).

This is the first report of development of a H_2_O_2_ sensor for female germline in *Drosophila*. The expression analysis suggest that this sensor could be used for detecting H_2_O_2_ in the germline cells. In fact, untreated germaria show roGFP2 excitation ratio profile close to that of reduced germarium indicating that normal physiological conditions are indeed reducing. Colocalization of roGFP2-Orp1 protein with ATP5α indicated that the protein is indeed targeted to the mitochondria. roGFP2-Orp1 expression in the GSCs was rather uncharacteristic when females were exposed to limited nutrients, rapamycin and chloroquine. It is well established that starvation induces oxidative stress (Filomeni et al., 2015). However, roGFP2-Orp1 fluorescence excitation did not shift from 488 to 405 nm in starved and CQ treated animals. In contrast, rapamycin treatment induced excitation shift of roGFP2-Orp1 which could be due to induction of mitochondrial remodeling, however, it needs further investigation (Chiao et al., 2016). Colocalization experiments with CathepsinL suggested that this sensor could also be used to monitor mitophagy in the germline cells. Thus, mito-roGFP2-Orp1 sensor could be used as a dual reporter for mitophagy and for measuring H_2_O_2_ production during oogenesis. We hope that these transgenic lines will provide a valuable tool that can be used in performing genetic screens that aid in identifying novel regulators of autophagy flux and redox homeostasis during oogenesis.

## Author Contributions

KN, NM, GS, and BS designed and performed the experiments. All authors wrote the manuscript and commented on it.

## Conflict of Interest Statement

The authors declare that the research was conducted in the absence of any commercial or financial relationships that could be construed as a potential conflict of interest.

## Supplementary Material

FIGURE S1 (A) Expression of nosP GFP-Ref(2)P nos 3′UTR in larval ovaries and fat body. Dotted line marks the boundary of larval ovary. Scale bar 10 μm. **(B)** Expression of nosP GFP-Ref(2)P nos 3′UTR in the germarium. The germarium is immunostained with anti-α-spectrin antibody. Arrows point to spectrosomes, arrowheads point to GFP-Ref(2)P punctae, asterisks mark the cap cells and the dotted line marks the GSCs. Scale bar 10 μm. **(C)** Whole ovaries of nosP GFP-Ref(2)P transgenic flies exposed to pharmacological conditions. Scale bar 500 μm. **(D)** GFP-Ref(2)P rod shaped structures pointed by arrow heads in germarium and a stage 6 egg chamber. Dotted ovals mark the GSCs. Scale bar 10 μm for germarium and 20 μm for egg chamber.

FIGURE S2 (A) nosP GFP-Ref(2)P nos 3′UTR transgenic immunostained for GFP. Dotted ovals mark the GSCs and asterisks mark the cap cells. Arrow heads point to merge of GFP-Ref(2)P and immunostained GFP. Inset shows enlarged region of punctae. Scale bar 10 μm. **(B)** nosP GFP-Ref(2)P nos 3′UTR transgenic immunostained for Ref(2)P. Dotted ovals mark the GSCs and asterisks mark the cap cells. Arrow heads point to merge of GFP-Ref(2)P and immunostained Ref(2)P. Inset shows enlarged region of punctae. Scale bar 10 μm. **(C)** Interleaved scatter graph showing Pearson’s coefficient as a measure of colocalization of GFP-Ref(2)P with immunostained GFP and immunostained Ref(2)P. Error bars represent SD in red and the mean is blue. *n* = 22 and *n* = 23 for anti-GFP and anti-Ref(2)P respectively. **(D)** Column graph showing the proportion of germarium with and without GFP-Ref(2)P punctae in fed conditions. Total of 94 germaria were analyzed.

FIGURE S3 (A) Expression of nosP mCherry-Atg8a nos 3′UTR in larval ovaries. Dotted line marks the boundary of larval ovary. Scale bar 10 μm. **(B)** Expression of nosP mCherry-Atg8a nos 3′UTR in the germarium. The germarium is immunostained with anti-α-spectrin antibody. Arrows point to spectrosomes, arrowheads point to mCherry-Atg8a punctae, asterisks mark the cap cells and the dotted line marks the GSCs. Scale bar 10 μm. **(C)** nosP mCherry-Atg8a nos 3′UTR transgenic flies subject to incrementally various days of starvation. Dotted ovals mark the GSCs. Scale bar 10 μm. **(D)** Interleaved scatter graph showing mCherry-Atg8a punctae per germarium upon starvation for incrementally various days. Error bars represent SD in red and the mean is blue. *n* = 8 for fed, *n* = 7 for 1, 2, 3 days and *n* = 13 for 4 days starvation respectively. ^∗∗^*p* < 0.01, ^∗∗∗^*p* < 0.001.

FIGURE S4 (A) nosP mCherry-Atg8a nos 3′UTR transgenic immunostained with anti-GABARAP antibody. Dotted ovals mark the GSCs. Scale bar 10 μm. **(B)** Comparison of UASp.mCherry.Atg8a and nosP mCherry-Atg8a lines. Germarium of UASp.mCherry.Atg8a; nosGal4VP16 and nosP mCherry-Atg8a transgenic subjected to fed and starved condition. Fed (6 days) and starved (2 days fed + 4 days starved). Dotted ovals mark the GSCs. Scale bar 10 μm. **(C)** Germarium of UASp.mCherry.Atg8a; nosGal4VP16 (left) and nosP mCherry-Atg8a (right) where mCherry-Atg8a punctae present in GSCs are pointed by arrowheads. Dotted ovals mark the GSCs. Scale bar 10 μm. **(D)** Interleaved scatter graph showing mCherry-Atg8a punctae in germarium of UASp.mCherry.Atg8a; nosGal4VP16 and nosP mCherry-Atg8a transgenic subjected to fed and starved condition. Fed (6 days) and starved (2 days fed + 4 days starved) ^∗∗^*p* < 0.01, ^∗∗∗^*p* < 0.001. **(E)** Interleaved scatter graph plotted for the mean intensity of mCherry-Atg8a in UASp.mCherry.Atg8a; nosGal4VP16 and nosP mCherry-Atg8a flies as a function of fed and starved condition. Fed (6 days) and starved (2 days fed + 4 days starved) ^∗∗∗^*p* < 0.001. For **(D,E)** 20 germarium were analyzed each for fed and starved for UASp.mCherry.Atg8a; nosGal4VP16. For nosP mCherry-Atg8a transgenic, 25 and 26 germarium were analyzed for fed (6 days) and starved (2 days fed + 4 days starved) respectively. Error bars represent SD in red and the mean is blue.

FIGURE S5 (A) Interleaved scatter graph showing mCherry-Atg8a punctae in germarium of nosP mCherry-Atg8a (control) flies and nosP mCherry-Atg8a in combination with RNAi for Atg8a. The number of germarium analyzed are 22 for control and 24 for Atg8a-RNAi. Error bars represent SD in red and the mean is blue. ^∗∗∗∗^*p* < 0.0001. **(B)** Rescue of male lethality of Atg8a^KG07569^ insertion mutant by nosP mCherry Atg8a. Schematic and genotypes of the cross performed for the rescue experiment along with number and proportion of viable F1 imagoes. Columns for rescued males are highlighted with red dotted boxes.

FIGURE S6 (A) Colocalization (yellow) of GFP-Ref(2)P (green) with CathepsinL (red) marked by arrow heads. Cathepsin-L marks the lysosomes. Arrow points to lysosome alone. Inset shows enlarged region of colocalization. Scale bar 20 μm. **(B)** mCherry-Atg8a can be used to visualize and distinguish between autophagosomes and autophagolysosomes. Arrow heads mark the autophagolysosomes (yellow) which are fusion of autophagosomes marked by mcherry-Atg8a (red) and lysosomes marked by Cathepsin-L (green). Arrows mark the autophagosomes. Inset shows enlarged region of colocalization. Scale bar 10 μm. **(C)** Stage 9 egg chamber of mCherry-Atg8a; GFP-Ref(2)P fly immunostained for Cathepsin-L. Inset shows enlarged region having a puncta (arrow) positive for GFP, mCherry as well as Cathepsin-L. Scale bar 10 μm.

FIGURE S7 Expression of nosP mito-roGFP2-Orp1 nos 3’UTR in larval ovaries. Dotted line marks the boundary of larval ovary. Scale bar 10 μm.

FIGURE S8 Western blot analysis using anti-GFP antibodies of ovarian extracts expressing GFP-Ref(2)P. 130 kDa GFP-Ref(2)P band could be detected in fed, starved and starved + CQ treated ovaries for 12 h **(A)** and 24 h **(B)**. Loading control actin is shown below for the same samples. Western blot analysis using anti-mCherry antibodies of ovarian extracts expressing mCherry-Atg8a. 44, 41, and 29 kDa bands corresponding to mCherry-Atg8a-I, mCherry-Atg8a-II and free mCherry respectively could be detected in fed, starved and starved + CQ treated ovaries for 12 h **(C)** and 24 h **(D)**. Loading control actin is shown below for the same samples.
