# Correction: Generation and characterization of germline-specific autophagy and mitochondrial reactive oxygen species reporters in *Drosophila*

**DOI:** 10.3389/fcell.2026.1900126

**Published:** 2026-07-28

**Authors:** Kiran Nilangekar, Nidhi Murmu, Govind Sahu, Bhupendra V. Shravage

**Affiliations:** 1 Developmental Biology Group, Agharkar Research Institute, Pune, India; 2 Department of Biotechnology, Savitribai Phule Pune University (SPPU), Pune, India

**Keywords:** GFP-Ref(2)P, mCherry-Atg8a, mito-roGFP2-Orp1, autophagic flux, redox, germline stem cell, autophagy, Atg8a

In the published article an error was made in the concentrations of the components of RIPA buffer.

A correction has been made to the section Material and Methods, Western Blotting:

The sentence “For lysate preparation, 25–30 flies were dissected in a buffer containing 1 M NaCl, 50 mM Tris and protease inhibitors” should read as “For lysate preparation, 25–30 flies were dissected in a buffer containing 150 mM NaCl, 25 mM Tris and protease inhibitors”.

The composition of RIPA buffer was previously stated as “1 M NaCl, 50 mM Tris, 1% Nonidet-P 40, 5% Sodium deoxycholate, 1% SDS”.

The corrected composition appears as follows; “150 mM NaCl, 25 mM Tris, 1% Nonidet-P 40, 0.5% Sodium deoxycholate, 0.1% SDS”.

The original version of this article has been updated.

