# Supplementary figures and images for: Generation and Characterization of Germline-Specific Autophagy and Mitochondrial Reactive Oxygen Species Reporters in Drosophila
Source: Front Cell Dev Biol. 2019 Apr 3;7:47. doi: 10.3389/fcell.2019.00047 (PMC6456670; doi:10.3389/fcell.2019.00047)

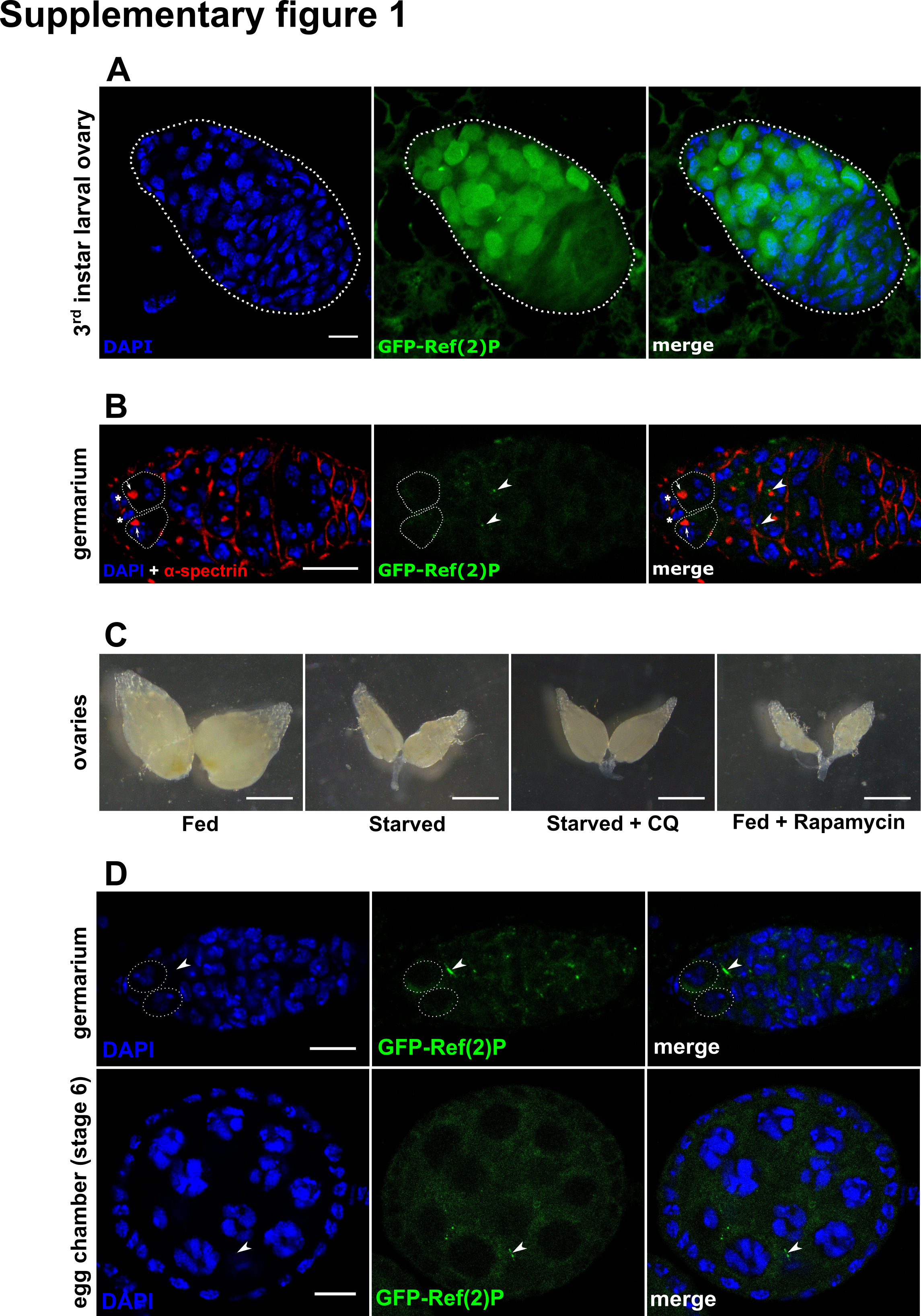

Supplement: FIGURE S1 — (A) Expression of nosP GFP-Ref(2)P nos 3′UTR in larval ovaries and fat body. Dotted line marks the boundary of larval ovary. Scale bar 10 μm. (B) Expression of nosP GFP-Ref(2)P nos 3′UTR in the germarium. The germarium is immunostained with anti-α-spectrin antibody. Arrows point to spectrosomes, arrowheads point to GFP-Ref(2)P punctae, asterisks mark the cap cells and the dotted line marks the GSCs. Scale bar 10 μm. (C) Whole ovaries of nosP GFP-Ref(2)P transgenic flies exposed to pharmacological conditions. Scale bar 500 μm. (D) GFP-Ref(2)P rod shaped structures pointed by arrow heads in germarium and a stage 6 egg chamber. Dotted ovals mark the GSCs. Scale bar 10 μm for germarium and 20 μm for egg chamber. [file Image_1.TIFF]

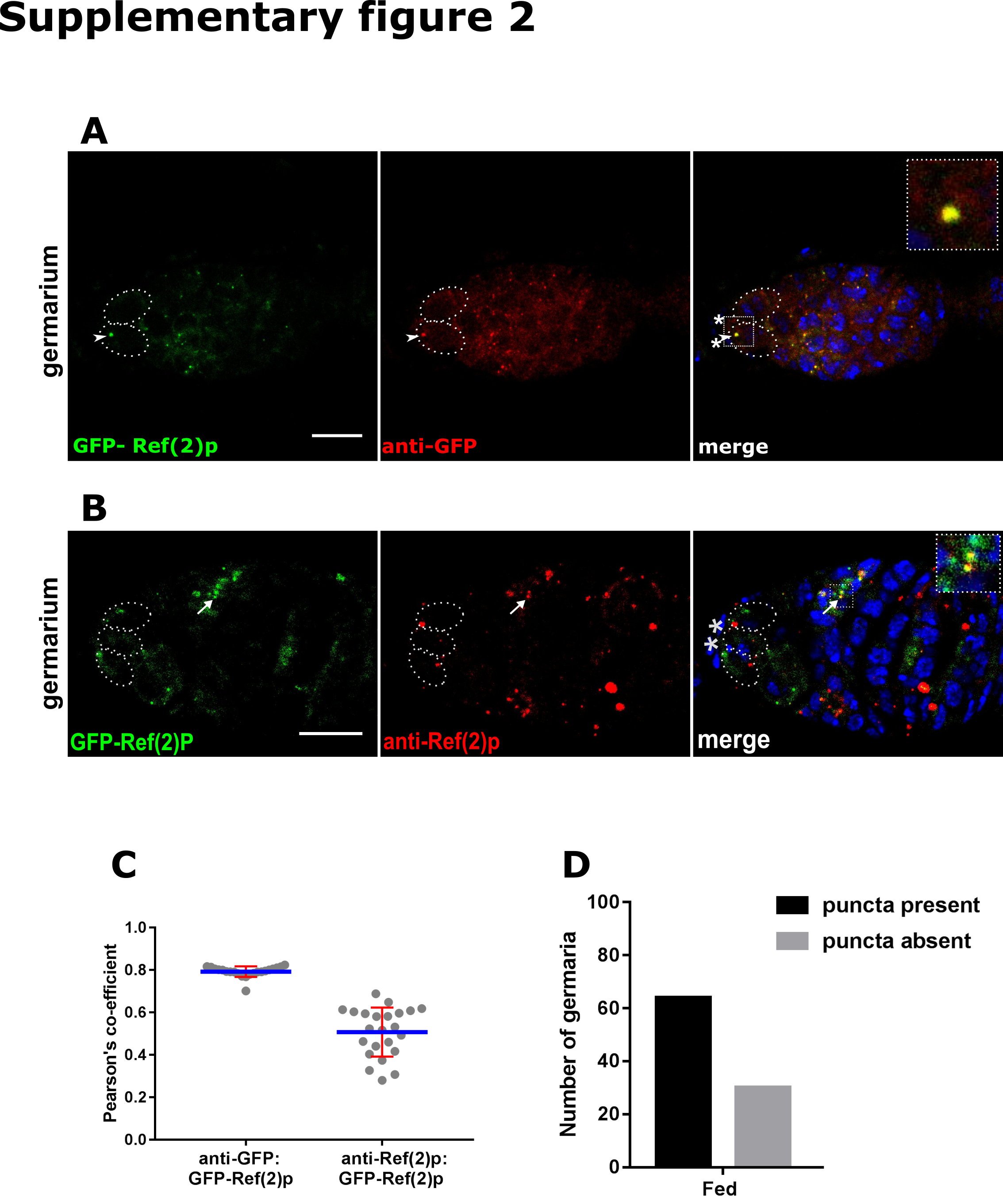

Supplement: FIGURE S2 — (A) nosP GFP-Ref(2)P nos 3′UTR transgenic immunostained for GFP. Dotted ovals mark the GSCs and asterisks mark the cap cells. Arrow heads point to merge of GFP-Ref(2)P and immunostained GFP. Inset shows enlarged region of punctae. Scale bar 10 μm. (B) nosP GFP-Ref(2)P nos 3′UTR transgenic immunostained for Ref(2)P. Dotted ovals mark the GSCs and asterisks mark the cap cells. Arrow heads point to merge of GFP-Ref(2)P and immunostained Ref(2)P. Inset shows enlarged region of punctae. Scale bar 10 μm. (C) Interleaved scatter graph showing Pearson’s coefficient as a measure of colocalization of GFP-Ref(2)P with immunostained GFP and immunostained Ref(2)P. Error bars represent SD in red and the mean is blue. n = 22 and n = 23 for anti-GFP and anti-Ref(2)P respectively. (D) Column graph showing the proportion of germarium with and without GFP-Ref(2)P punctae in fed conditions. Total of 94 germaria were analyzed. [file Image_2.TIFF]

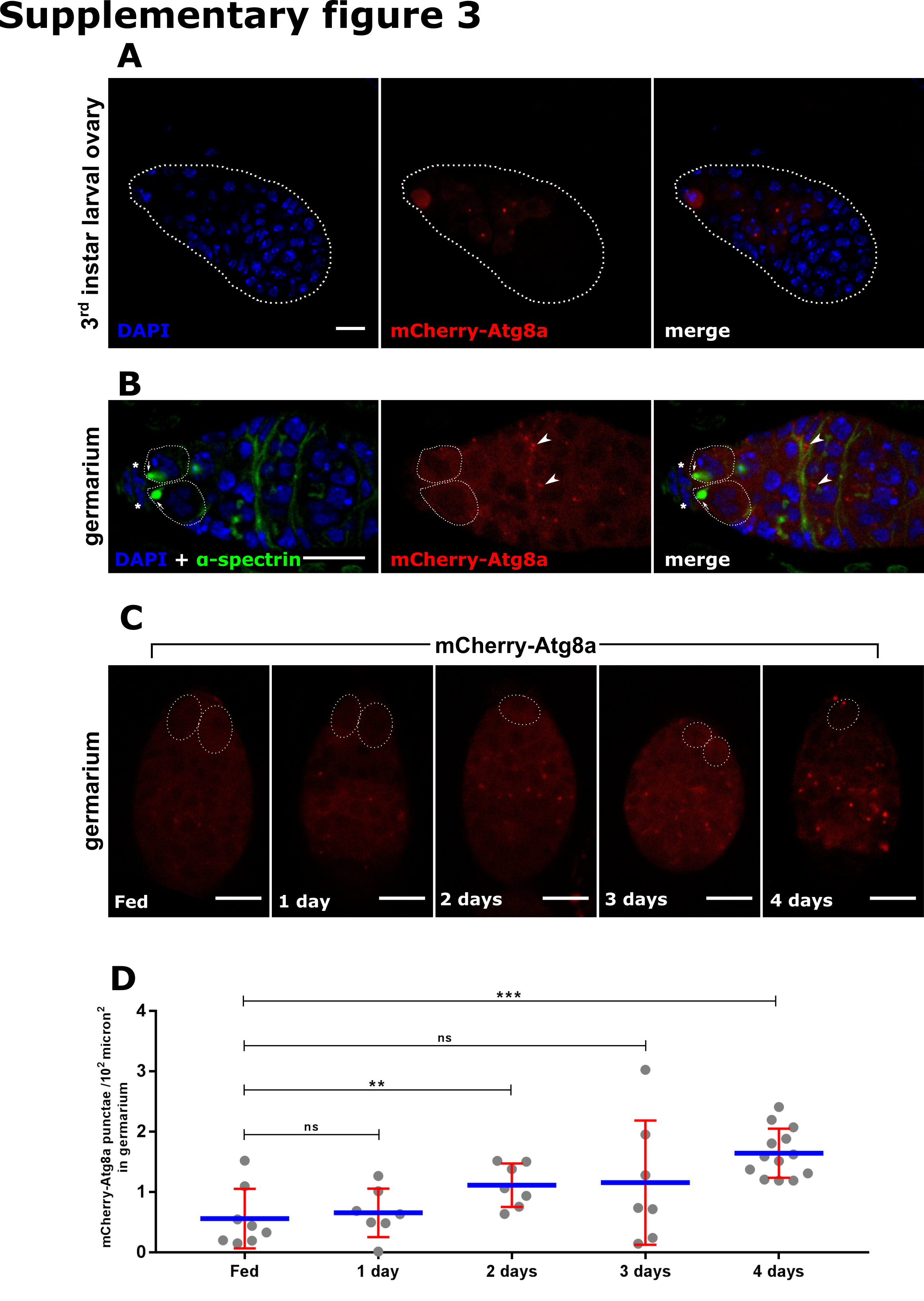

Supplement: FIGURE S3 — (A) Expression of nosP mCherry-Atg8a nos 3′UTR in larval ovaries. Dotted line marks the boundary of larval ovary. Scale bar 10 μm. (B) Expression of nosP mCherry-Atg8a nos 3′UTR in the germarium. The germarium is immunostained with anti-α-spectrin antibody. Arrows point to spectrosomes, arrowheads point to mCherry-Atg8a punctae, asterisks mark the cap cells and the dotted line marks the GSCs. Scale bar 10 μm. (C) nosP mCherry-Atg8a nos 3′UTR transgenic flies subject to incrementally various days of starvation. Dotted ovals mark the GSCs. Scale bar 10 μm. (D) Interleaved scatter graph showing mCherry-Atg8a punctae per germarium upon starvation for incrementally various days. Error bars represent SD in red and the mean is blue. n = 8 for fed, n = 7 for 1, 2, 3 days and n = 13 for 4 days starvation respectively. ∗∗p < 0.01, ∗∗∗p < 0.001. [file Image_3.TIFF]

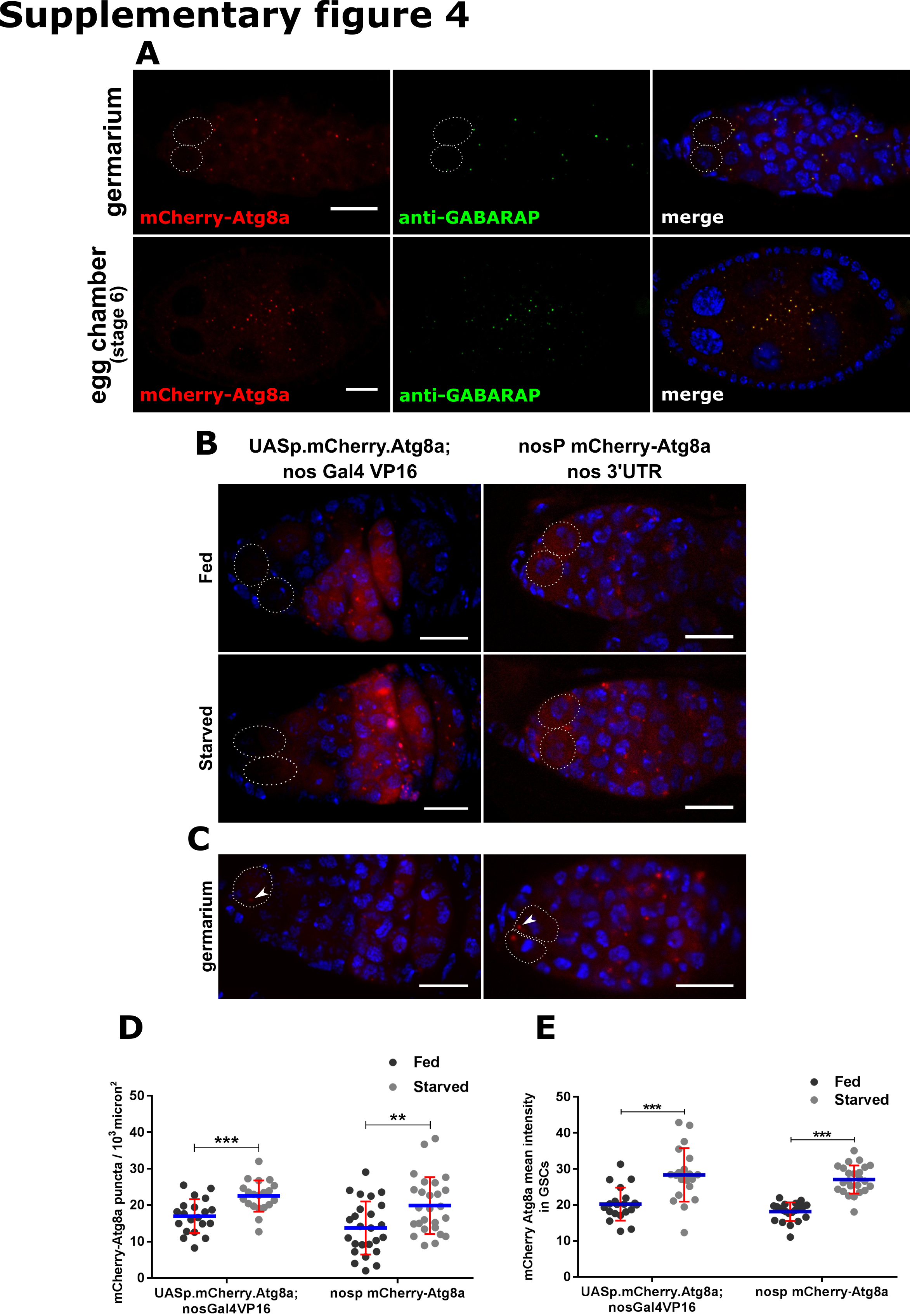

Supplement: FIGURE S4 — (A) nosP mCherry-Atg8a nos 3′UTR transgenic immunostained with anti-GABARAP antibody. Dotted ovals mark the GSCs. Scale bar 10 μm. (B) Comparison of UASp.mCherry.Atg8a and nosP mCherry-Atg8a lines. Germarium of UASp.mCherry.Atg8a; nosGal4VP16 and nosP mCherry-Atg8a transgenic subjected to fed and starved condition. Fed (6 days) and starved (2 days fed + 4 days starved). Dotted ovals mark the GSCs. Scale bar 10 μm. (C) Germarium of UASp.mCherry.Atg8a; nosGal4VP16 (left) and nosP mCherry-Atg8a (right) where mCherry-Atg8a punctae present in GSCs are pointed by arrowheads. Dotted ovals mark the GSCs. Scale bar 10 μm. (D) Interleaved scatter graph showing mCherry-Atg8a punctae in germarium of UASp.mCherry.Atg8a; nosGal4VP16 and nosP mCherry-Atg8a transgenic subjected to fed and starved condition. Fed (6 days) and starved (2 days fed + 4 days starved) ∗∗p < 0.01, ∗∗∗p < 0.001. (E) Interleaved scatter graph plotted for the mean intensity of mCherry-Atg8a in UASp.mCherry.Atg8a; nosGal4VP16 and nosP mCherry-Atg8a flies as a function of fed and starved condition. Fed (6 days) and starved (2 days fed + 4 days starved) ∗∗∗p < 0.001. For (D,E) 20 germarium were analyzed each for fed and starved for UASp.mCherry.Atg8a; nosGal4VP16. For nosP mCherry-Atg8a transgenic, 25 and 26 germarium were analyzed for fed (6 days) and starved (2 days fed + 4 days starved) respectively. Error bars represent SD in red and the mean is blue. [file Image_4.TIFF]

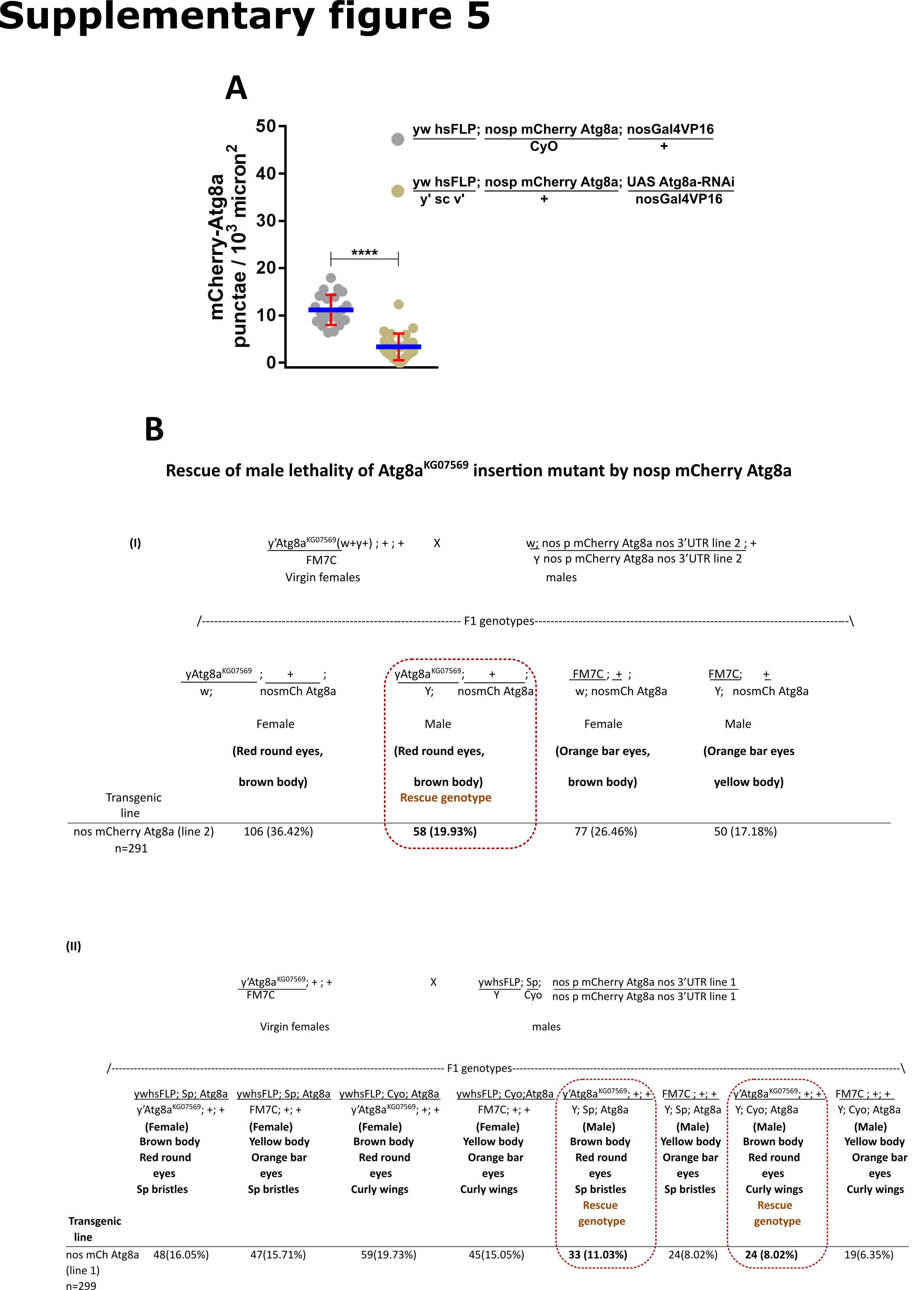

Supplement: FIGURE S5 — (A) Interleaved scatter graph showing mCherry-Atg8a punctae in germarium of nosP mCherry-Atg8a (control) flies and nosP mCherry-Atg8a in combination with RNAi for Atg8a. The number of germarium analyzed are 22 for control and 24 for Atg8a-RNAi. Error bars represent SD in red and the mean is blue. ∗∗∗∗p < 0.0001. (B) Rescue of male lethality of Atg8aKG07569 insertion mutant by nosP mCherry Atg8a. Schematic and genotypes of the cross performed for the rescue experiment along with number and proportion of viable F1 imagoes. Columns for rescued males are highlighted with red dotted boxes. [file Image_5.TIFF]

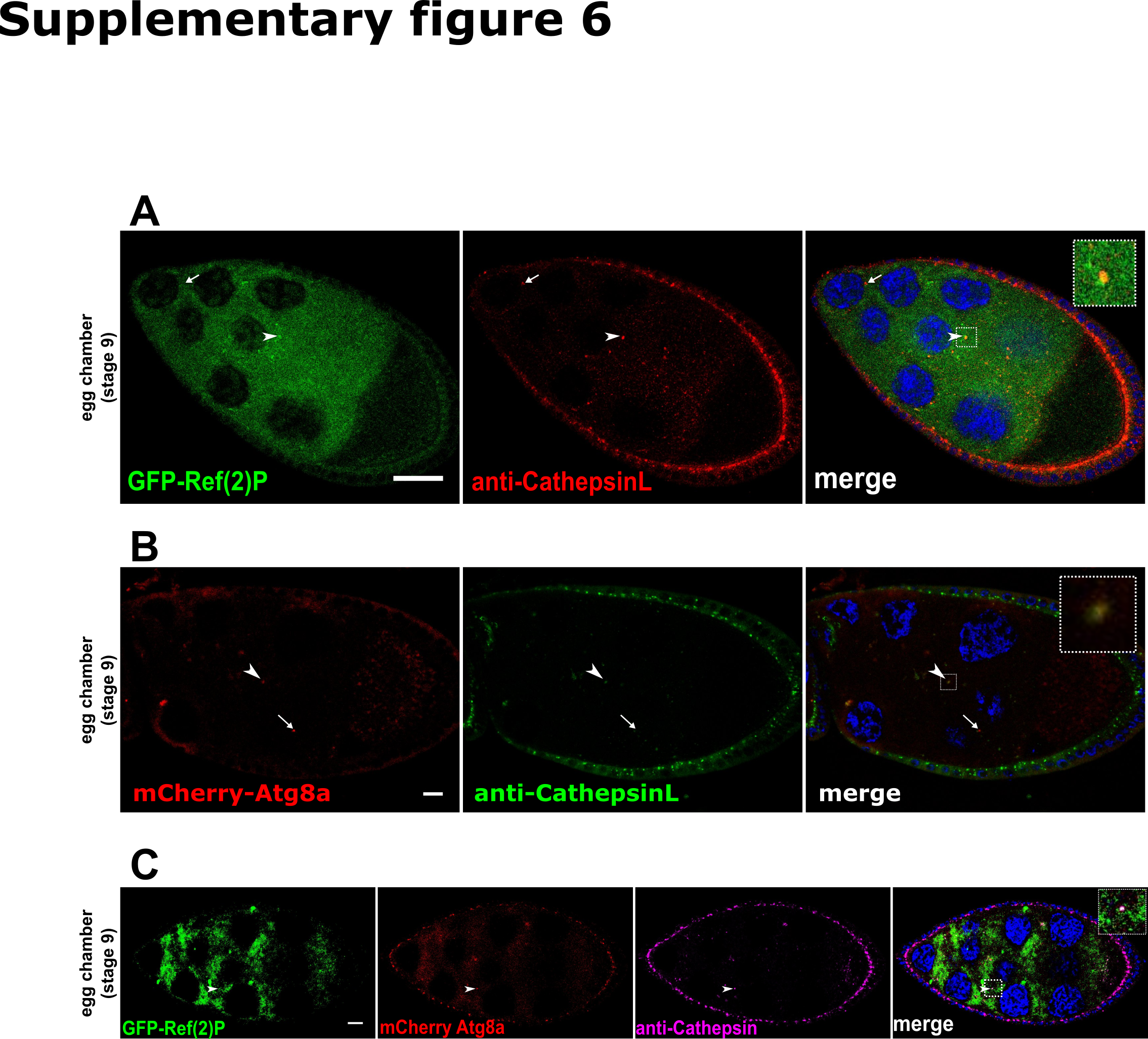

Supplement: FIGURE S6 — (A) Colocalization (yellow) of GFP-Ref(2)P (green) with CathepsinL (red) marked by arrow heads. Cathepsin-L marks the lysosomes. Arrow points to lysosome alone. Inset shows enlarged region of colocalization. Scale bar 20 μm. (B) mCherry-Atg8a can be used to visualize and distinguish between autophagosomes and autophagolysosomes. Arrow heads mark the autophagolysosomes (yellow) which are fusion of autophagosomes marked by mcherry-Atg8a (red) and lysosomes marked by Cathepsin-L (green). Arrows mark the autophagosomes. Inset shows enlarged region of colocalization. Scale bar 10 μm. (C) Stage 9 egg chamber of mCherry-Atg8a; GFP-Ref(2)P fly immunostained for Cathepsin-L. Inset shows enlarged region having a puncta (arrow) positive for GFP, mCherry as well as Cathepsin-L. Scale bar 10 μm. [file Image_6.TIFF]

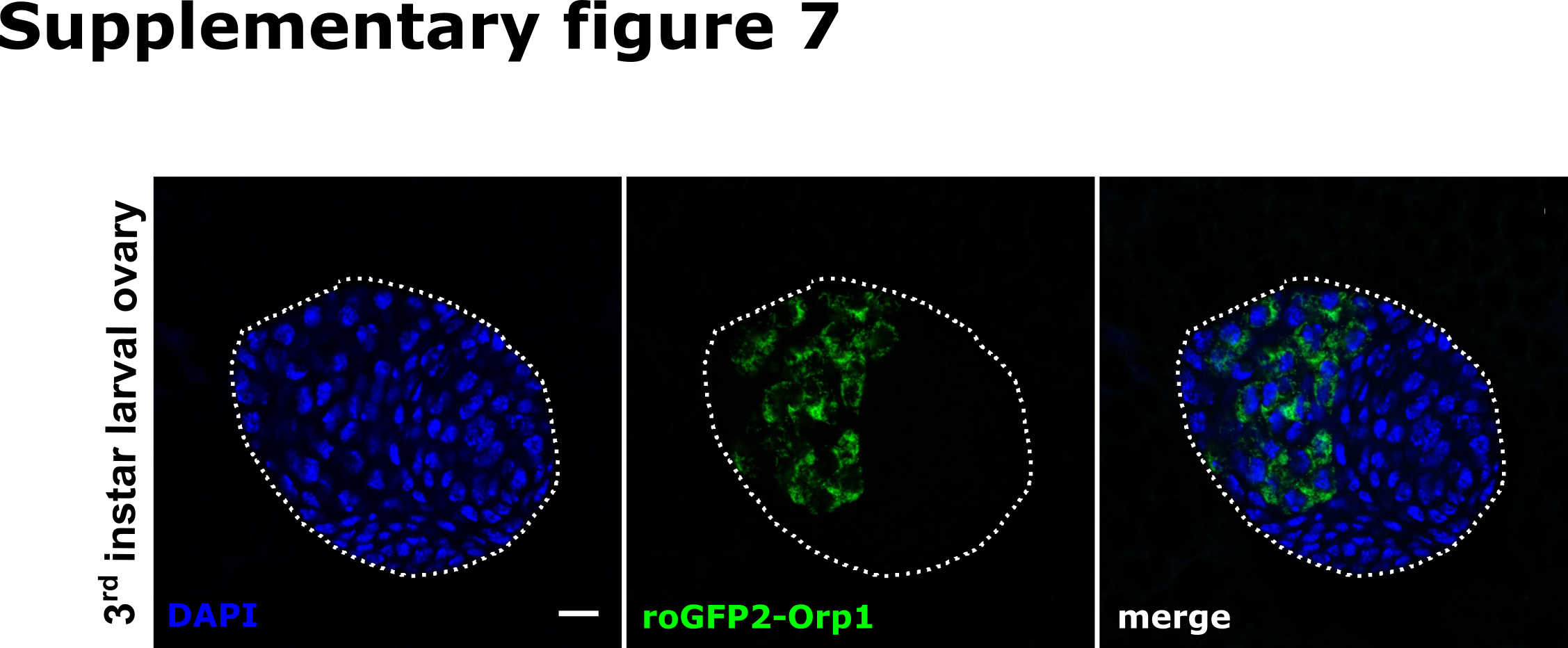

Supplement: FIGURE S7 — Expression of nosP mito-roGFP2-Orp1 nos 3’UTR in larval ovaries. Dotted line marks the boundary of larval ovary. Scale bar 10 μm. [file Image_7.tiff]

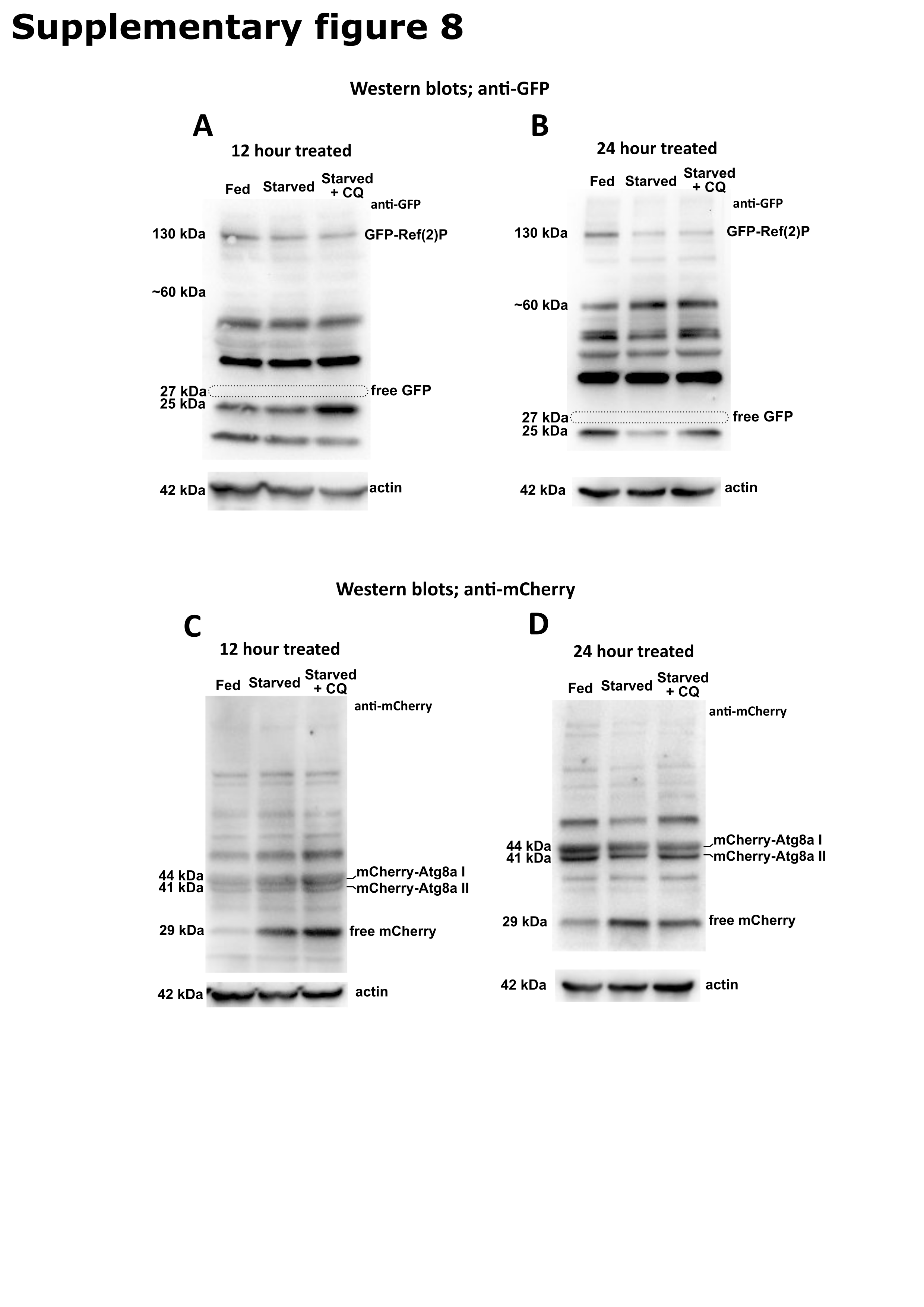

Supplement: FIGURE S8 — Western blot analysis using anti-GFP antibodies of ovarian extracts expressing GFP-Ref(2)P. 130 kDa GFP-Ref(2)P band could be detected in fed, starved and starved + CQ treated ovaries for 12 h (A) and 24 h (B). Loading control actin is shown below for the same samples. Western blot analysis using anti-mCherry antibodies of ovarian extracts expressing mCherry-Atg8a. 44, 41, and 29 kDa bands corresponding to mCherry-Atg8a-I, mCherry-Atg8a-II and free mCherry respectively could be detected in fed, starved and starved + CQ treated ovaries for 12 h (C) and 24 h (D). Loading control actin is shown below for the same samples. [file Image_8.TIFF]
